# Effect of a Novel Alpha/Beta Hydrolase Domain Protein on Tolerance of *K. marxianus* to Lignocellulosic Biomass Derived Inhibitors

**DOI:** 10.3389/fbioe.2020.00844

**Published:** 2020-07-24

**Authors:** Dan Wu, Dongmei Wang, Jiong Hong

**Affiliations:** ^1^School of Life Sciences, University of Science and Technology of China, Hefei, China; ^2^Hefei National Laboratory for Physical Sciences at the Microscale, Hefei, China

**Keywords:** lignocellulose, multiple inhibitors, tolerance, thioesterase, esterase, mitochondria, *Kluyveromyces marxianus*

## Abstract

The multiple inhibitors tolerance of microorganism is important in bioconversion of lignocellulosic biomass which is a promising renewable and sustainable source for biofuels and other chemicals. The disruption of an unknown α/β hydrolase, which was termed KmYME and located in mitochondria in this study, resulted in the yeast more susceptible to lignocellulose-derived inhibitors, particularly to acetic acid, furfural and 5-HMF. The *KmYME* disrupted strain lost more mitochondrial membrane potential, showed increased plasma membrane permeability, severer redox ratio imbalance, and increased ROS accumulation, compared with those of the non-disrupted strain in the presence of the same inhibitors. The intracellular concentration of ATP, NAD and NADP in the *KmYME* disrupted strain was decreased. However, disruption of KmYME did not result in a significant change of gene expression at the transcriptional level. The KmYME possessed esterase/thioesterase activity which was necessary for the resistance to inhibitors. In addition, KmYME was also required for the resistance to other stresses including ethanol, temperature, and osmotic pressure. Disruption of two possible homologous genes in *S. cerevisiae* also reduced its tolerance to inhibitors.

## Graphical Abstract

Possible tolerance mechanisms to inhibitors of KmYME in *K. marxianus*.

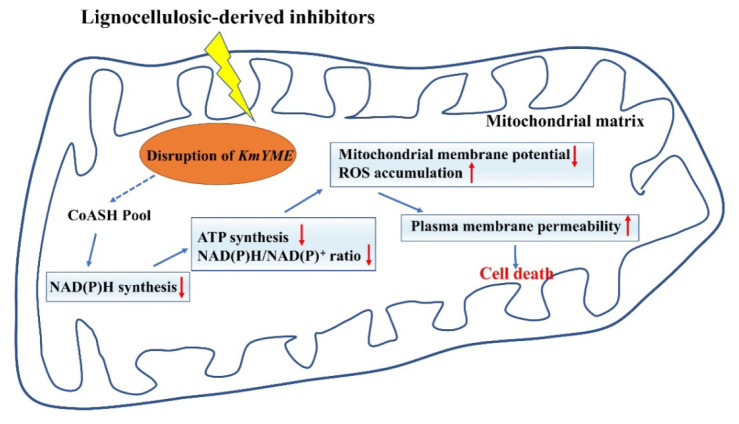

## Introduction

Lignocellulosic biomass is deemed as a promising resource for renewable biofuels and other chemicals due to its low cost, large-scale availability, and non-competition with food production (Kamm and Kamm, 2004; Yinbo et al., 2006; Somerville et al., 2010). However, during lignocellulosic biomass pretreatment, inhibitors are generated as by-products of sugar and lignin degradation, including weak acids, furan derivatives and phenolic compounds. These compounds have different toxic mechanisms and synergistic effects that inhibit microbial cell metabolism and fermentation (Palmqvist and Hahn-Hägerdal, 2000; Almeida et al., 2007). Hence, determining the mechanism for tolerance to these inhibitors is important in the construction of a robust strain for industrial fermentation.

*Kluyveromyces marxianus* is a ‘generally regarded as safe’ (GRAS) microorganism that has attracted increasing attention in bioethanol fermentation of lignocellulosic biomass due to its thermo-tolerance, high growth rate, and broad substrate spectrum (Zhang et al., 2015). *K. marxianus* strains can grow with a growth rate of 0.86–0.99 h^–1^ at 40°C, and some strains even grow at temperatures as high as 52°C (Banat and Marchant, 1995). Elevated temperature is suitable for cellulolytic enzymes used in lignocellulose saccharification (optimal temperature 45–50°C) and provides advantages for the simultaneous saccharification and fermentation (SSF), as well as simultaneous saccharification and co-fermentation (SSCF) (Zhang et al., 2016; Kong et al., 2019). Moreover, industrial fermentation at elevated temperature reduces cooling cost and risk of contamination (Banat and Marchant, 1995). Xylose is one of the main hydrolysis products of lignocellulosic biomass, aside from glucose, and *K. marxianus* can natively utilize xylose (Wilkins et al., 2008). However, the tolerance of *K. marxianus* to multiple inhibitors is not very strong and the knowledge of *K. marxianus* tolerance to multiple inhibitors was scarce in previous studies (Oliva et al., 2003; Oliva et al., 2004).

In our previous study, the expression of an uncharacterized α/β-hydrolase domain (ABHD)-containing gene (GenBank BAP69980, locus tag KMAR_10772) of *K. marxianus* was significantly up-regulated in the presence of inhibitors derived from the pretreatment of lignocellulosic biomass (Wang et al., 2018). Here we focused on its effect on tolerance to inhibitors, the protein property and its inhibitor tolerance mechanism. We found that the disruption of this gene reduces the tolerance to inhibitors in *K. marxianus*. This unknown protein was identified to be located in the mitochondria matrix and possessed the esterase/thioesterase activity *in vitro* which was necessary for the resistance to inhibitors. We inferred that the disruption of this gene would reduce the tolerance to inhibitors by disturbing the intracellular CoASH pool which interferes with ATP and NAD(P)H synthesis in the presence of inhibitors. In addition, KmYME showed a broad spectrum of resistance to other stresses, including osmic pressure (sugar and salt), ethanol and temperature. The homologous protein in *Saccharomyces cerevisiae* also showed resistance on multiple inhibitors. This study may provide useful information for better understanding resistance mechanism of multiple inhibitors, which is important in the creation of a robust strain for industrial fermentation that can use cellulosic biomass as a substrate.

## Materials and Methods

### Culture and Microorganisms

Yeast extract/peptone dextrose (YPD) medium containing 10 g/L yeast extract, 20 g/L peptone, 20 g/L glucose was used to culture yeast. Synthetic dropout (SD) medium containing 6.7 g/L yeast nitrogen base without amino acids, 20 g/L glucose supplemented with appropriate supplements were used to screen yeast transformants. Luria–Bertani (LB) medium with 100 μg/mL ampicillin was used to culture *Escherichia coli* Top10 for gene cloning and BL21 (DE3) for protein expression. *K. marxianus* NBRC1777 was obtained from the NITE Biological Resource Center (Tokyo, Japan). *K. marxianus* YHJ010 is the *TRP1*, *LEU2*, *URA3* auxotrophic strain of NBRC1777 (Hong et al., 2007). *S. cerevisiae* W303 1A is the *ADE2*, *TRP1*, *LEU2*, *URA3*, and *HIS3* auxotrophic strain (ATCC 208352).

### Plasmid Construction

All of the plasmids used in this study are listed in Table 1. Plasmid construction is described in Additional File 1 in detail. Primer pairs are listed in Additional File 1: Supplementary Table S1.

**TABLE 1:** Plasmids used in this study.

**Plasmids**	**Selection marker and description**	**References**
YEGAP	*ScTRP1*, P*_ScGAPDH_*, T*_ScGAPDH_*	Hong et al., 2001
YEUKmPGK	*ScURA3*, P*_KmPGK_*, T*_ScGAPDH_*	Yang et al., 2015
pMD18T-Δ ScURA3	*Amp*^R^, no function *ScURA3*	Zhang et al., 2016
pCF.1676	*Amp*^R^, pJK148- ase1P*-klp3(1-335)-TagRFP-tom22	Li et al., 2015
pCG	*Amp*^R^, CBM-EGFP- pTWIN1	Hong et al., 2008
pWD001	*Amp*^R^, *KmYME*-T vector	This study
pWD002	*Amp*^R^, *KmYME* inserted with *ScURA3*	This study
pWD003	*Amp*^R^, *ScTRP1*, P*_ScGAPDH_*- *KmYME* -T*_ScGAPDH_*	This study
pWD004	*Amp*^R^, *ScURA3*, P*_KmPGK_*-*EGFP*- T*_ScGAPDH_*	This study
pWD005	*Amp*^R^, *ScURA3*, P*_KmPGK_*- *KmYME* -*EGFP*-T*_ScGAPDH_*	This study
pWD006	*Amp*^R^, *ScTRP1*, P*_ScGAPDH_*-*KmCox* -*RFP*-T*_ScGAPDH_*	This study
pWD007	*Amp*^R^, *ScURA3*, P*_KmPGK_*- *KmYME* (1–20 aa)-*EGFP* -T*_ScGAPDH_*	This study
pWD008	*Amp*^R^, *ScURA3*, P*_KmPGK_*- *KmYME* (1–40 aa)-*EGFP* -T*_ScGAPDH_*	This study
pWD009	*Amp*^R^, *ScURA3*, P*_KmPGK_*- *KmYME* (41–360 aa)-*EGFP* -T*_ScGAPDH_*	This study
pWD010	*Amp*^R^, *KmYME* expressed under P*_T7_*	This study
pWD024	*Amp*^R^, *NP_011545* inserted with *ScURA3*	This study
pWD025	*Amp*^R^, *NP_011529* inserted with *ScURA3*	This study
pWD026	*Amp*^R^, *ScURA3*, P*_KmPGK_*-*KmCox* -*RFP*-T*_ScGAPDH_*	This study
pWD032	*Amp*^R^, *ScTRP1*, P*_ScGAPDH_*- *KmYME* (GYSLG→GHSMG) -T*_ScGAPDH_*	This study
pWD033	*Amp*^R^, *ScTRP1*, P*_ScGAPDH_*- *KmYME* (GYSLG→AYALA) -T*_ScGAPDH_*	This study
pWD034	*Amp*^R^, *KmYME* (GYSLG→GHSMG) expressed under P*_T7_*	This study
pWD035	*Amp*^R^, *KmYME* (GYSLG→AYALA) expressed under P*_T7_*	This study

Briefly, pWD002, pWD024, and pWD025 were constructed for the disruption cassette of *KmYME*, *NP_011545* and *NP_011529*, respectively. pWD004 was constructed for the expression of EGFP; pWD005 and pWD006 were constructed for the expression of KmYME-EGFP and KmCox-RFP in the KmYME localization assay. pWD007, pWD008 and pWD009 were constructed for the expression of the truncated KmYME (1–20 aa)-EGFP, KmYME (1–40 aa)-EGFP and KmYME (41–360 aa)-EGFP to identify the mitochondrial signal peptide. pWD026 was constructed for the expression of KmCox-RFP with a URA3 label to observe the mitochondrial morphology of the *KmYME* disrupted and non-disrupted strains. pWD010, pWD034 (GYSLG→GHSMG from pWD010) and pWD035 (GYSLG→AYALA from pWD010) were constructed for the expression of KmYME and KmYME mutants under the *T*_7_ promoter in *E. coli* BL21 (DE3) cells. pWD003, pWD032 (GYSLG→GHSMG from pWD003) and pWD033 (GYSLG→AYALA from pWD003) were used to overexpress KmYME and the KmYME mutants under the *ScGAPDH* promoter in *K. marxianus.*

### Strain Construction

All of the yeast strains used in this study are listed in Table 2 and illustrated in Additional File 1: Supplementary Figure S3. Transformation was conducted using the lithium acetate method (Abdel-Banat et al., 2010). The transformants were screened on SD medium with appropriate supplements and confirmed by PCR using genomic DNA as a template. The detailed strain construction is described in Additional File 1.

**TABLE 2:** Yeast strains used in this study.

**Strains**	**Relevant genotype**	**References**
YHJ010	*K. marxianus*, Δ*KmURA3*:: *KAN^R^, ΔKmLEU2*::*HISG, ΔKmTRP1*::*HISG*	Hong et al., 2007
W303 1A	*S. cerevisiae* ATCC 208352, *MATa, ade2-1, his3-11, 15, leu2-3, 112, trp1-1, ura3-1, can1-100*	ATCC 208352
YWD001	*K. marxianus*, YHJ010, Δ*KmYME*::*ScURA3*	This study
YWD002	*K. marxianus*, YHJ010, pWD003, *KmYME*	This study
YWD003	*K. marxianus*, YWD001, *ScTRP1*	This study
YWD004	*K. marxianus*, YWD001, pWD003, *KmYME*	This study
YWD005	*K. marxianus*, YHJ010, *ScURA3*	This study
YWD009	*K. marxianus*, YHJ010, *ScTRP1*	This study
YWD010	*K. marxianus*, YWD005, *ScTRP1*	This study
YWD021	*K. marxianus*, YHJ010, pWD004, *EGFP*	This study
YWD022	*K. marxianus*, YHJ010, pWD005, *KmYME*-*EGFP*	This study
YWD024	*K. marxianus*, YWD021, pWD006, *KmCox* -*RFP*	This study
YWD026	*K. marxianus*, YWD022, pWD006, *KmCox* -*RFP*	This study
YWD028	*K. marxianus*, YHJ010, pWD007, *KmYME* (1–20 aa)-*EGFP*	This study
YWD030	*K. marxianus*, YHJ010, pWD008, *KmYME* (1–40 aa)-*EGFP*	This study
YWD032	*K. marxianus*, YHJ010, pWD009, *KmYME* (41–360 aa)-*EGFP*	This study
YWD034	*S. cerevisiae* W303 1A, Δ*NP_011545*::*ScURA3*	This study
YWD036	*S. cerevisiae* W303 1A, Δ*NP_011529*::*ScURA3*	This study
YWD037	*S. cerevisiae* W303 1A, YWD034, Δ *ScURA3*	This study
YWD038	*S. cerevisiae* W303 1A, YWD037, Δ*NP_011529*::*ScURA3*	This study
YWD040	*S. cerevisiae* W303 1A, *ScURA3*	This study
YWD046	*K. marxianus*, YWD003, Δ *ScURA3*, pWD026, *KmCox* -*RFP*	This study
YWD047	*K. marxianus*, YWD004, Δ *ScURA3*, pWD026, *KmCox* -*RFP*	This study
YWD048	*K. marxianus*, YWD010, Δ *ScURA3*, pWD026, *KmCox* -*RFP*	This study
YWD051	*S. cerevisiae* W303 1A, YWD038, *ScTRP1*	This study
YWD052	*S. cerevisiae* W303 1A, YWD038, pWD003, *KmYME*	This study
YWD053	*S. cerevisiae* W303 1A, YWD040, *ScTRP1*	This study
YWD074	*K. marxianus*, YWD001, pWD032, *KmYME* (GYSLG→GHSMG)	This study
YWD076	*K. marxianus*, YWD001, pWD033, *KmYME* (GYSLG→AYALA)	This study

### Samples Preparation and Transcriptome Analysis

#### Cultivation Conditions

*Kluyveromyces marxianus* YWD001 and YWD005 were pre-cultivated in 5 mL of YPD medium at 42°C overnight. Then, the cells were transferred into 500-mL Erlenmeyer flasks containing 100 mL of YPD medium with an initial OD_600_ of 0.5 and cultivated at 42°C with shaking at 250 rpm in an orbital shaker until the OD_600_ = 6 (the early exponential phase of growth). The cells were then incubated without or in the presence of the inhibitor mixture containing 5.3 g/L acetate acid, 1.3 g/L furfural, 1.3 g/L HMF, and 0.5 g/L phenols (4-hydroxybenzaldehyde, syringaldehyde, catechol and vanillin with 0.13 g/L of each compound, pH 6.0) for 1 h. We chose these inhibitors because they are the main inhibitors produced during lignocellulosic biomass pretreatment (Taherzadeh and Karimi, 2011). Yeast cells were then recovered and stored at −80°C until RNA isolation.

### *RNA-Seq* Analysis

Total RNA of each sample was extracted and cDNA libraries were prepared as previously described (Wang et al., 2018). The resulting cDNA library products were then shotgun sequenced (101 bp paired-end read) with the Illumina HiSeq 4000 instrument (Illumina, San Diego, CA, United States) using a customer sequencing service (Majorbio Co., Ltd, Shanghai, China).

Annotation and bioinformatics analysis were performed as previously reported (Wang et al., 2018). Clean reads were mapped to the reference genomic sequence of *K. marxianus* NBRC1777 from GenBank with accession No. AP014599–AP014607 (Inokuma et al., 2015) using TopHat^1^. In addition, information from the DEGs was subjected to GO and KEGG significant enrichment analyses to identify biological functions and metabolic pathways in which these genes participated. For differential gene expression analysis, reads or fragments per kilobase of exon model per million mapped reads (FPKM) was used as a value of normalized gene expression. Genes were considered differentially expressed in a given library when *p*-value < 0.05 and a greater than two-fold change in expression across libraries was observed.

### Extraction of RNA and qPCR Analysis

Total RNA was isolated using a yeast total RNA extraction kit (Sangon Biotech Co. Shanghai, China). Isolated RNA was treated with RNase-free DNase I (Toyobo, Osaka, Japan) and cDNA was synthesized using the ReverTra Ace qPCR RT Master Mix kit (Toyobo, Osaka, Japan) as described (Wang et al., 2018). Real-time PCR was conducted on a Bio-Rad iCycler iQ (Bio-Rad, Hercules, CA, United States) using the THUNDERBIRD SYBR qPCR mix kit (Toyobo, Osaka, Japan). The primers for *KmYME* and the *ACT1* internal control are shown in Additional File 1: Supplementary Table S1. The cycle threshold values (*C*_T_) were determined and the relative fold differences were calculated using the 2^–Δ^ ^Δ^ *^*C*^*^*T*^ method (Nolan et al., 2006) with *ACT1* as the endogenous reference gene. The fold change was shown as the sign of the log_2_ transformed fold change (FC) values (log_2_ FC).

### Fluorescence Microscopy

Localization of KmYME in *K. marxianus* was conducted as described previously (Mo et al., 2016). Imaging was conducted using a Perkin Elmer spinning-disk confocal microscope (PerkinElmer, Inc., Norwalk, CT, United States) equipped with a Zeiss PlanApo 100X/1.4 NA objective and a Photometrics EMCCD camera Evolve 51234. Images were captured by the DeltaVision softWoRx software (Applied Precision) and processed by deconvolution and z-stack projection. All images were analyzed using ImageJ.

### Mitochondrial Protein Separation and Extraction

Purification of mitochondria was performed as previously described (Gregg et al., 2009). Briefly, cells were harvested at 3000 × *g* for 5 min, washed twice with ddH_2_O, resuspended in DTT buffer [100 mM Tris/H_2_SO_4_ (pH 9.4), 10 mM dithiothreitol] and shaken for 20 min at 37°C. The cells were recovered and resuspended in snailase solution [20 mM potassium phosphate (pH 6.0), 1.2 M sorbitol and 40 mg/g (wet weight cells) snailase]. After 1 h incubation at 37°C, the cells were harvested and resuspended in ice-cold homogenization buffer [10 mM Tris/HCl (pH 7.4), 0.6 M sorbitol, 1 mM EDTA, 0.2% (w/v) BSA]. The cells were disrupted by sonication in an ice bath until half of the cell membranes were broken. Subsequently, mitochondria were isolated by differential centrifugation.

Second, the mitochondrial outer-membrane proteins and mitoplast (inner-membrane plus matrix) proteins were isolated by adding 0.15 mg/mL digitonin (Sangon Biotech Co. Shanghai, China) and vigorously agitated for 15 min using a vortex mixer. Then, the mixture was centrifuged at 10,000 × *g* for 30 min at 4°C. The resulting supernatant was the outer-membrane protein fraction and the pellet was the mitoplast protein (inner-membrane plus matrix) fraction (Nishimura et al., 2014).

### Western Blotting

Western blotting was performed as described previously (Nishimura et al., 2014). Anti-GFP antibody and anti-RFP antibody were purchased from YEASEN Biotech Co., Shanghai, China.

### Preparation of the Samples for the Measurement of Intracellular ATP, NAD(P)^+^, NAD(P)H, MMP, Plasma Membrane Permeability, Mitochondria Morphology and ROS Detection

Cells were cultivated in YPD medium until the OD_600_ = 6, then they were recovered and incubated for another 2 h without inhibitors, or in the presence of the inhibitor mixture (5.3 g/L acetate acid, 1.3 g/L furfural, 1.3 g/L HMF, and 0.5 g/L phenols), 3 g/L acetic acid, 6 g/L furfural+6 g/L 5-HMF, or 2 g/L phenols, respectively at 42°C. The cells were collected by centrifugation for 5 min at 3000 × *g* for the detection of intracellular ATP and NAD(P)^+^, NAD(P)H, MMP, plasma membrane permeability, mitochondria morphology and intracellular ROS level.

### Intracellular ATP Extraction and Quantification

Half milliliter of cells were vortexed for 3 min in 0.5 mL 5% cold trichloroacetic acid to extract ATP. Then the samples were centrifuged at 10,000 × *g* for 10 min at 4°C. The resulting supernatant was diluted 1000 times with 0.05 mol/L Tris-acetate (pH 7.8). The amount of ATP was determined using the ENLITEN ATP Assay System Bioluminescence kit (Promega Corporation, Madison, WI, United States) following the manufacturer’s instruction. At OD_600_ = 1, the concentration of the cells was equivalent to 0.411 g/L dry cell weight (DCW) (Zhang et al., 2013).

### Quantification of the Intracellular NAD(P)^+^ and NAD(P)H

A 5-mL sample of yeast culture was withdrawn and sprayed into quenching solution (60% methanol and 70 mM HEPES). Then, the intracellular coenzymes NAD(P)H and NAD(P)^+^ were extracted and quantified using an EnzyChrom^TM^ NAD(P)^+^/NAD(P)H assay kit (BioAssay Systems, Hayward, CA, United States) following the manufacturer’s instruction (Wang et al., 2018).

### Rhodamine 123 (Rh123) and Propidium Iodide (PI) Double Fluorescent Staining

After washing twice with phosphate buffered saline (PBS), 100 μL yeast cells were collected and suspended in 10 mM 2-morpholinoethanesulfonic acid buffer (MES) containing 0.1 mM MgCl_2_ and 2% (w/v) glucose. Then, Rh123 (YEASEN Biotech Co., Shanghai, China) was added to a final concentration of 10 μM and the sample was incubated for 20 min at 37°C in the dark. After the sample was washed twice with PBS, PI (YEASEN Biotech Co., Shanghai, China) was added to a final concentration of 100 μg/mL and the sample was incubated for 1 min at 37°C in the dark. At last, the cells were resuspended in 1 mL PBS and immediately examined by CytoFLEX flow cytometry (Beckman Coulter, Inc., United States). The excitation wavelength was set to 480 nm, the application of side scatter (SSC) and forward scatter (FSC) were linearly amplified, and a logarithmic amplification was performed for fluorescence channel FL1 (FITC) and FL3 (PC5.5). The results were analyzed using the FlowJo software.

### ROS Assay

Cells were washed twice with PBS, then resuspended in PBS at a final concentration of 10^7^ cells/mL with the addition of 10 μg 2′,7′-dichlorofluorescein diacetate (DCFH-DA) (Sigma, United States) (Wang et al., 2015). After incubation at 37°C for 60 min, the cells were washed twice with PBS. The fluorescence (excitation, 488 nm; emission, 525 nm) was detected using a CytoFLEX flow cytometer. The results were analyzed using the FlowJo software.

### Intracellular CoASH and Acetly-CoA Detection

After the cells were recovered, the intracellular CoASH and acetly-CoA detection were performed as described previously (Boynton et al., 1994) by high performance liquid chromatography (HPLC) equipped with C18 column (4.6 mm × 250 mm, 5 μm Phenomenex, Los Angeles, CA, United States) and UV detector (Diode Array Detector, Agilent, Palo Alto, CA, United States).

### Recombinant Expression and Purification of KmYME and Its Mutants

To recombinantly express KmYME and its mutants, *E. coli* BL21 (DE3) was transformed with the plasmids pWD010, pWD034, or pWD035 (Table 1) and the resulting strains were cultivated in a 1 L shaking flask containing 400 mL LB medium with 100 μg/mL ampicillin at 37°C until OD_600_ = 0.6–0.8. Then, expression was induced with 0.5 mM IPTG at 37°C for 4 h, the cells were recovered and lysed in lysate buffer (20 mM Tris-HCl pH 7.8, 0.3 M NaCl) by sonication (Sonics Vibra cell Model VCX130, SONICS & MATERIALS INC. Newtown, CT, United States). After centrifugation, recombinant KmYME was purified from the lysate supernatant using a nickel-sepharose resin column (TransGen Biotech Co., Beijing, China) following the manufacturer’s instruction. The obtained protein was analyzed by SDS-PAGE. Protein concentration was determined using a Modified Bradford Protein Assay Kit (Sangon Biotech Co. Shanghai, China).

### Enzymatic Activity Assays

One unit of activity is defined as the amount of enzyme required to hydrolyze 1 μmol of substrate in 1 min. The enzymes were assayed as follows. The thioesterase activity was assayed as described by McMahon and Prather (2014). In brief, the enzyme was incubated with 100 μM acyl-CoA, 1 mM DTNB [5,5′-Dithiobis-(2-nitrobenzoic acid)] (Sangon Biotech Co. Shanghai, China) in 100 mM HEPES, pH 8.0 at 37°C for 5 min. The progress of the reaction was monitored through the change in absorbance at 412 nm using the molar extinction coefficient of 5-thio-2-nitrobenzoate (14,150 M^–1^ cm^–1^), which is formed when DTNB reacts with free CoA. Acetyl-CoA(C2-CoA), butyryl-CoA(C4-CoA), succinyl-CoA and decanoyl-CoA (C10-CoA) (Sigma, United States) were used as substrates.

Esterase activity was assayed as described by Kademi et al. (1999). In brief, the enzyme was incubated with 100 μM pNP aliphatic esters in 50 mM Tris-HCl, pH 8.0 at 37°C for 5 min. The amount of produced pNP was calculated in absorbance at 410 nm. pNPC2), *p*-nitrophenyl butyrate (pNPC4) or *p*-nitrophenyl decanoate (pNPC10) (Sigma, United States) were used as substrates.

## Results

### *KmYME* Was Significantly Up-Regulated in the Presence of Lignocellulosic-Derived Inhibitors

**FIGURE 1:**
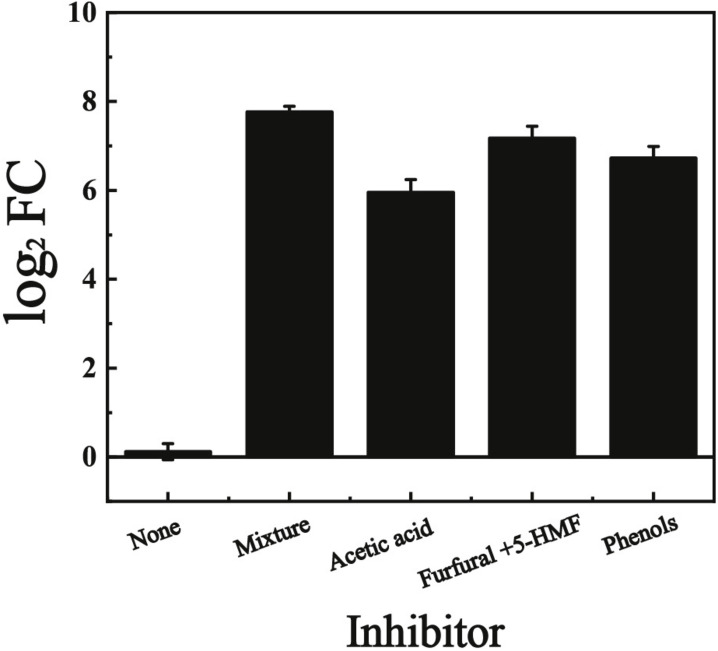
qPCR results of *KmYME* expression in YHJ010 cells with or without lignocellulose-derived inhibitors. All values are the means of three biological replicates ± standard deviation.

In our previous study, the gene *KMAR_10772*, encoding an uncharacterized ABHD-containing protein was significantly up-regulated when the cells were cultivated with multiple inhibitors (acetate acid, furfural, HMF, and phenols) (Wang et al., 2018). In this study, this gene was named *KmYME* and its expression in YHJ010 cells (Table 2) in the presence of each single kind of inhibitor (2.5 g/L acetic acid, 1.5 g/L furfural + 1.5 g/L 5-HMF, or 1.0 g/L phenols (4-hydroxybenzaldehyde, syringaldehyde, catechol, and vanillin), respectively) at 42°C was also determined by quantitative real-time PCR (qPCR) (Figure 1). The change of expression was shown as the log_2_ fold change (FC) (log_2_ FC). As shown in Figure 1, *KmYME* was up-regulated with a log_2_ FC value of 5.95 ± 0.29, 7.17 ± 0.27, and 6.73 ± 0.26, corresponding to acetic acid, furfural + 5-HMF, and phenols, respectively, compared with the log_2_ FC value of 0.12 ± 0.18 with no inhibitor. Therefore, regardless of the presence of the inhibitor mixture or a single inhibitor, the expression of *KmYME* was enhanced.

### Disruption of *KmYME* Reduced the Tolerance to Inhibitors in *K. marxianus*

**FIGURE 2:**
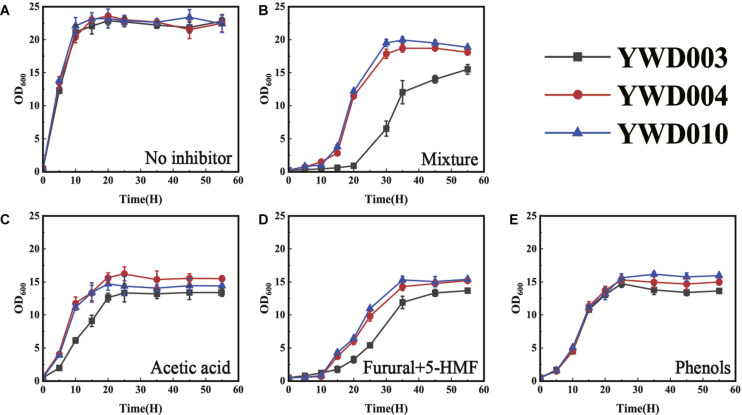
Growth of YWD003, YWD004, and YWD010 in YPD containing **(A)** no inhibitor, **(B)** inhibitor mixture, **(C)** acetic acid, **(D)** furfural + 5-HMF, or **(E)** phenols. All values are the means of three biological replicates ± standard deviation at each of the time points.

After the significantly up-regulated expression of *KmYME* was confirmed, its effect on tolerance to inhibitors was evaluated through gene disruption and retro-complementation. As shown in Figure 2A, when cultivated at 42°C in YPD medium without inhibitors, the growth of YWD003 (*KmYME* disrupted strain) was similar to those of YWD004 (*KmYME* retro-complemented strain) and YWD010 (YHJ010 complemented with URA3 and TRP1, as a control) (Table 2). With the inhibitor mixture or each single inhibitor, however, the growth of all strains was repressed with a longer lag phase, slower growth rate, and less final biomass yield (OD_600_) (Figures 2B–E).

Specifically, when the cells were cultured in YPD medium with the inhibitor mixture (3.0 g/L acetate acid, 0.7 g/L furfural, 0.7 g/L HMF, and 0.28 g/L phenols) (Figure 2B), 2.5 g/L acetic acid (pH 4) (Figure 2C) or 1.5 g/L furfural + 1.5 g/L HMF (Figure 2D), respectively, the lag phase of YWD003 was longer than that of YWD004 and YWD010; and the exponential phase maximum growth rate (h^–1^) of YWD003 (0.20 ± 0.01, 0.27 ± 0.02, 0.13 ± 0.07) was slower than that of YWD004 (0.31 ± 0.01, 0.41 ± 0.02, 0.33 ± 0.02) and YWD010 (0.28 ± 0.01, 0.41 ± 0.01, 0.33 ± 0.00). The biomass yield (OD_600_ maximum) of YWD003 was also less than that of YWD004 and YWD010. However, the difference of the growth in the presence of 1.0 g/L phenols was not obvious among those three strains except that the biomass yield (OD_600_ maximum) of YWD003 was a little lower than that of YWD004, and YWD010 (Figure 2E).

Afterward, tolerance to inhibitors of *KmYME* overexpressed strains (YWD002) (Table 2) was also evaluated by cultivating the cells with or without inhibitors. The results indicated that overexpression *KmYME* did not improve *K. marxianus* tolerance to inhibitors (Additional File 2: Supplementary Figure S1).

Moreover, the growth of YWD003, YWD004 and YWD010 in synthetic dropout medium (SD) was also determined. The tolerance of these strains to inhibitors all decreased and the strains only grew under lower concentrations of inhibitors. YWD003 again showed a worse performance than YWD004 and YWD010 in the presence of the inhibitor mixture (Additional File 2: Supplementary Figure S2).

Because acetic acid in the medium reduces the pH and acetic acid is often produced during yeast fermentation (Cordente et al., 2013), a synergistic effect of acetate and pH was determined. As shown in Additional File 2: Supplementary Figure S3, the growth of YWD003 showed a worse performance than YWD004 and YWD010 in YPD with acetate (pH 6). Also, the acetate and pH showed a synergistic effect on the inhibition of *K. marxianus* growth. Disruption of *KmYME* led the strain (YWD003) to be more sensitive to acetate and a low pH.

### The KmYME Protein Was Located in the Mitochondrial Mitoplast

**FIGURE 3:**
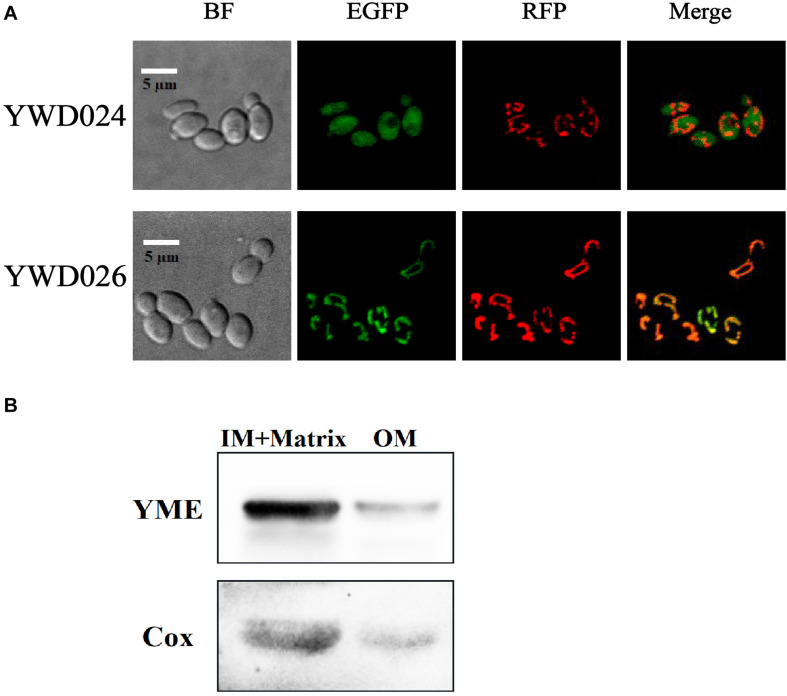
Analysis of the location of KmYME. **(A)** Subcellular localization. YWD024: expressing EGFP and KmCox-RFP. YWD026: expressing KmYME-EGFP and KmCox-RFP. KmCox-RFP was used as a mitochondria marker. **(B)** Intra-mitochondrial localization by western blotting. IM + Matrix, inner-membrane and matrix fraction of mitochondria; OM, outer-membrane fraction of mitochondria.

To determine the roles of KmYME in the tolerance to inhibitors, the intracellular localization of KmYME was investigated. KmYME with an EGFP fused at its C-terminus (KmYME-EGFP) was expressed in strain YWD022 (Table 2) to determine its intracellular location. These data suggested that the fusion protein was located in the mitochondria. Therefore, KmCox (GenBank: XP_022674814), a subunit of cytochrome oxidase on the inner mitochondrial membrane (Bottinger et al., 2013), was used to co-localize the position of KmYME. KmCox-RFP, as a mitochondria marker, was co-expressed with KmYME-EGFP in strain YWD026 (Table 2). KmCox-RFP was expressed in the EGFP-expressing strain YWD024 as a control (Table 2). As shown in Figure 3A, KmYME was expressed in mitochondria and co-localized with KmCox-RFP. Subsequently, a series of truncated (1–20, 1-40, or 41–360 aa) or full length (1–360 aa) KmYME constructs were expressed with EGFP to determine the mitochondrial signal sequence of KmYME. The results indicated that the mitochondrial targeting sequence of KmYME was within 1–40 aa of the N- terminus, but after the first 20 aa (Additional File 2: Supplementary Figure S4).

To clarify the function of KmYME, a more accurate position in the mitochondria was determined using western blotting. The mitochondria of YWD026, which co-expressed KmCox-RFP and KmYME-EGFP was extracted for analysis. KmCox regarded as mitochondrial mitoplast (inner-membrane and matrix) indicator. As shown in Figure 3B, most of the KmYME-EGFP and KmCox-RFP was present in the fraction containing the mitochondrial mitoplast. Since there was no transmembrane domain found in KmYME using informatics analysis, KmYME was possibly a matrix protein.

### Disruption of *KmYME* Led to Decreased Intracellular ATP Concentrations in the Presence of Inhibitors

Many mitochondrial matrix proteins are involved in energy metabolism and ATP is the main source of energy for most cellular processes (Quijano et al., 2015). Hence, intracellular ATP levels were determined to evaluate if the disruption of *KmYME* affected ATP production. As shown in Table 3, without inhibitors, the intracellular ATP concentration of YWD003 [2.84 ± 0.23 μmol/g DCW (dry cell weight)], YWD004 (2.86 ± 0.06 μmol/g DCW), YWD010 (2.96 ± 0.28 μmol/g DCW) were similar. With the addition of inhibitors, however, the ATP concentration in all strains obviously decreased. Specifically, the ATP concentration of YWD003 in the presence of the inhibitor mixture, acetic acid, and furfural+5-HMF was 0.36 ± 0.08 μmol/g DCW, 0.63 ± 0.03 μmol/g DCW, and 0.60 ± 0.12 μmol/g DCW, respectively, which was obviously less than those of YWD004 (0.68 ± 0.09 μmol/g DCW, 1.22 ± 0.18 μmol/g DCW, and 1.01 ± 0.10 μmol/g DCW, respectively) and YWD010 (0.74 ± 0.03 μmol/g DCW, 1.12 ± 0.10 μmol/g DCW, and 0.99 ± 0.04 μmol/g DCW, respectively). These results indicated that disruption of *KmYME* led to a significant decrease of intracellular ATP concentration. However, the difference in intracellular ATP concentration was not so obvious among YWD003 (0.82 ± 0.10 μmol/g DCW), YWD004 (0.98 ± 0.13 μmol/g DCW) and YWD010 (0.89 ± 0.10 μmol/g DCW) in the presence of phenols (Table 3), which was consistent with the growth analysis (Figure 2). In addition, the intracellular ATP concentration decreased with 2 h treatment of 10 μM oligomycin, while there was no obvious difference among strains YWD003 (0.58 ± 0.05 μmol/g DCW), YWD004 (0.62 ± 0.07 μmol/g DCW) and YWD010 (0.64 ± 0.12 μmol/g DCW).

**TABLE 3:** Intracellular ATP concentration (μmol/g DCW).

**Inhibitor**	**Oligomycin**	**None**	**Mixture**	**Acetic acid**	**Furfural +5-HMF**	**Phenols**
YWD003	0.58 ± 0.05	2.84 ± 0.23	0.36 ± 0.08	0.63 ± 0.03	0.60 ± 0.12	0.82 ± 0.10
YWD004	0.62 ± 0.07	2.86 ± 0.06	0.68 ± 0.09	1.22 ± 0.18	1.01 ± 0.10	0.98 ± 0.13
YWD010	0.64 ± 0.12	2.96 ± 0.28	0.74 ± 0.03	1.12 ± 0.10	0.99 ± 0.04	0.89 ± 0.10

*All values are the means of three biological replicates ± standard deviation.*

### Disruption of *KmYME* Led to Decreased Intracellular NAD and NADP Concentrations in the Presence of Inhibitors

NAD (including NAD^+^ and NADH) and NADP (including NADP^+^ and NADPH) as a cofactor are key players in the energy and antioxidant system, and the redox balances of the NAD and NADP pool dictate metabolic processes (Ying, 2008). Therefore, the intracellular concentration of NAD (NAD^+^ + NADH) and NADP (NADP^+^ + NADPH) and NAD(P)H/NAD(P)^+^ ratio were determined.

**FIGURE 4:**
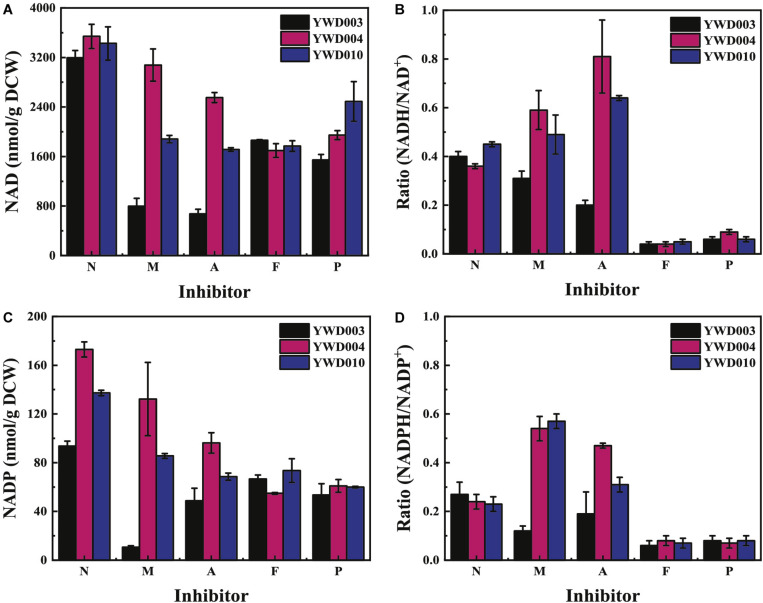
The intracellular concentration of the NAD and NADP coenzymes without inhibitors (N), and in the presence of the inhibitor mixture (M), acetic acid **(A)**, furfural + 5-HMF (F), or phenols (P). **(A)** NAD (NAD^+^+ NADH) concentration, **(B)** Ratio of NADH/NAD^+^, **(C)** NADP (NADP^+^+NADPH) concentration, **(D)** Ratio of NADPH/NADP^+^. All values are the means of three biological replicates ± standard deviation.

As shown in Figure 4A, without inhibitors (N), the intracellular NAD (NAD^+^ + NADH) concentrations in YWD003, YWD004, and YWD010 were similar. However, with the addition of inhibitors, the intracellular NAD concentration of all strains decreased. The NAD concentration of YWD003 decreased the most, with only 24.9 ± 3.1% remaining in the presence of the inhibitor mixture (M) and 21.0 ± 1.6% remaining in the presence of acetic acid (A) compared to the concentrations without inhibitors (N). The next was YWD010, the NAD concentration was 55.0 ± 2.6% in the presence of the inhibitor mixture (M) and 50.0 ± 3.0% in the presence of acetic acid (A) compared to the concentrations without inhibitors (N). The NAD concentration of YWD004 remained at 87.3 ± 12.0% in the presence of the inhibitor mixture (M) and 72.2 ± 1.7% in the presence of acetic acid (A) compared to the concentrations without inhibitors (N) (Figure 4A). However, in the presence of phenols (P), the intracellular NAD concentration of YWD003 was only slightly lower than that of YWD004 and YWD010, while in the presence of furfural+5-HMF (F), the NAD concentration of the three strains decreased to a similar degree (Figure 4A).

As shown in Figure 4B, the ratios of NADH/NAD^+^ in YWD003, YWD004, and YWD010 were similar without inhibitors (N). In the presence of the inhibitor mixture (M) or acetic acid (A), the ratio of NADH/NAD^+^ in YWD003 decreased, whereas the ratios in YWD004 and YWD010 increased. However, in the presence of phenols (P) or furfural+5-HMF (F), the ratio of NADH/NAD^+^ in all three strains obviously decreased.

The intracellular concentration of NADP (NADP^+^ + NADPH) and the ratio of NADPH/NADP^+^ were also determined. As shown in Figure 4C, without inhibitors, the intracellular NADP concentration of YWD003 was lower than the other two strains. Though the intracellular concentration of NADP was 20-fold lower than that of NAD, the pattern of the change in the presence of the various inhibitors was similar to that of NAD for all three strains; the change in the ratios of NADPH/NADP^+^ were also similar to the ratios of NADH/NAD^+^ (Figure 4D).

### Disruption of *KmYME* Injured the Integrity of the Plasma Membrane, Reduced the MMP and Increased Intracellular ROS Accumulation in the Presence of Inhibitors

Mitochondrial function, a key indicator of cell health, can be assessed by monitoring changes in the mitochondrial membrane potential (MMP) (Perry et al., 2011) using Rhodamine 123 (Rh123), a positively charged molecule that can accumulate in energized mitochondria. Decline of the MMP will cause leakage of Rh123 from the mitochondria, resulting in the decline of green fluorescence intensity (Rh123^–^) (H. Hong and Liu, 2004). Propidium iodide (PI) was used to measure cell plasma membrane integrity. This dye can enter the damaged membranes of dead cells to bind to DNA and produces red fluorescence (Graça da Silveira et al., 2002). Therefore, the permeabilized plasma membrane of a dead cell will result in higher red fluorescence intensity (PI^+^). In our study, the MMP and plasma membrane integrity of cells in response to various inhibitors was determined with Rh123 and PI double staining using flow cytometry to evaluate mitochondrial function and cell death.

**FIGURE 5:**
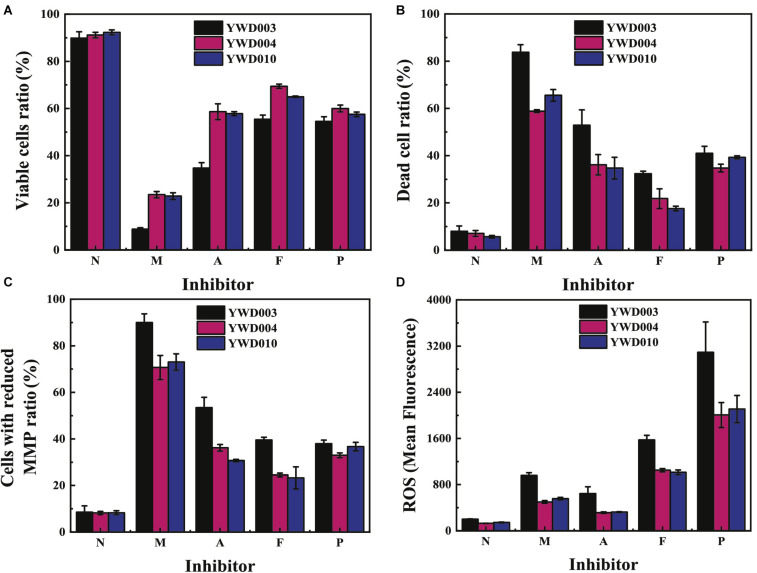
The flow cytometry results of **(A)** viable cells (Rh123^+^/PI^–^), **(B)** dead cells (PI^+^), **(C)** cells with reduced MMP (Rh123^–^), and **(D)** ROS levels in YWD003, YWD004, YWD010 without inhibitors (N), or in the presence of the inhibitor mixture (M), acetic acid (A), furfural and 5-HMF (F), or phenols (P).

As shown in Figure 5, compared with those without inhibitors, the ratio of viable cells (Rh123^+^/PI^–^) (Figure 5A) decreased while the percentage of dead cells (PI^+^) (Figure 5B) or cells with reduced MMP (Rh123^–^) (Figure 5C) of all strains increased in the presence of various inhibitors. Notably, in the presence of the inhibitor mixture (M), acetic acid (A) or furfural + 5-HMF (F), the ratio of viable cells of YWD003 (8.8 ± 0.6%; 34.8 ± 2.3%; 55.4 ± 1.8%, respectively) was much lower than that of YWD004 (23.5 ± 1.3%; 58.6 ± 3.4%; 69.4 ± 0.9%, respectively) and YWD010 (22.8 ± 1.5%; 57.8 ± 0.8%; 65.0 ± 0.3%, respectively); a higher ratio of dead cells or cells with reduced MMP was detected in strain YWD003 compared to that of strains YWD004 and YWD010. In the presence of phenols (P), the ratio of viable cells, dead cells (PI^+^) or cells with reduced MMP in YWD003 was not significantly different from those in YWD004 and YWD010. These results indicated that disruption of *KmYME* reduced the MMP and increased the permeability of the plasma membrane in the presence of acetic acid and furfural+5-HMF, which then caused loss of mitochondrial function and cell death.

In addition, in the presence of inhibitors, the morphology of the mitochondria in *K. marxianus* obviously changed. As shown in Additional File 2: Supplementary Figure S5, mitochondria in all three strains were fissured into small, short and round shapes and some mitochondria appeared swollen in the presence of various inhibitors, compared with those reticula form of mitochondria in the cells without inhibitors, suggesting that the mitochondria were impaired by the inhibitors. However, there was no obvious difference among strains YWD046 (*KmYME* disrupted strain), YWD047 (*KmYME* retro-complemented strain) and YWD048 (YHJ010 complemented with URA3 and TRP1, as a control). Even the intracellular concentration of NAD(P) and ATP were different under the same conditions. It is possible that the inhibitors had a strong ability to disrupt the mitochondrial morphology, but the difference was not obvious enough to be detected by microscopy.

Mitochondria is the main source of reactive oxygen species (ROS) in eukaryotes. Next, levels of intracellular ROS were determined using 2′,7′-dichlorofluorescein diacetate (DCFH-DA) staining to determine if disruption of *KmYME* could influence ROS accumulation in the presence of multiple inhibitors. As shown in Figure 5D, ROS accumulation obviously increased in the presence of various inhibitors. The levels of ROS in YWD003 were the highest in the presence of all inhibitors, compared with the levels in the other two strains, indicating that disruption of *KmYME* improved the accumulation of intracellular ROS in response to the presence of the inhibitors. It is noteworthy that the pattern of the amount of dead cells was not consistent with that of ROS accumulation (Figures 5B,D). Though the percent of dead cells was the highest in the presence of the mixture of inhibitors and the second highest was in the presence of acetic acid, the highest levels of ROS accumulation was induced by the presence of phenols, and the second highest levels by the presence of furfural+5-HMF, indicating that phenols and furfural+5-HMF may play a leading role among the inhibitors in ROS accumulation.

### Determination of the Enzyme Activity of the KmYME Protein

*KmYME* is described as an uncharacterized ABHD-containing protein YGR015C in GenBank. The ABHD superfamily includes proteases, lipases, esterases, dehalogenases, peroxidases, and epoxide hydrolases. Most of the homologous proteins of *KmYME* from other organisms are uncharacterized proteins. In this study, the *KmYME* gene was expressed in *Escherichia coli* BL21 (DE3) cells and the recombinant enzyme was purified. The enzyme was tested for the following activities: peroxidase, acetylcholinesterase (AChE), esterase or thioesterase using H_2_O_2_, 2-mercaptoethyl-trimethylammonium iodide acetate, C4-CoA and *p*-nitrophenyl butyrate (pNPC4) as substrates, respectively. As a result, KmYME could hydrolyze C4-CoA and pNPC4 but could not hydrolyze H_2_O_2_ and 2-mercaptoethyl-trimethylammonium iodide acetate. Thus, the KmYME protein was provisionally identified as an esterase and a thioesterase.

### Enzymatic Properties of KmYME and Its Mutants

The consensus pentapeptide GXSXG is found in virtually all lipases/esterases and generally contains the active site serine (Pérez et al., 2012) (Additional File 2: Supplementary Figure S6). In KmYME and ABHD11, a mammalian homolog, the conserved amino acid residues were GYSLG and GHSMG, respectively. Therefore, the amino acid residues GYSLG in the KmYME protein were substituted with GHSMG or AYALA. Then, the KmYME and its mutants were recombinantly expressed in *E. coli* BL21 (DE3) cells and purified (Additional File 2: Supplementary Figure S7). The enzymes were characterized using various substrates with different length carbon chains (Table 4). For pNP aliphatic ester substrates, the activity of KmYME was 2303.61 ± 154.69 nmol/min/mg and 600.59 ± 5.90 nmol/min/mg for *p*-nitrophenyl acetate (pNPC2) and pNPC4, respectively. There was no activity detected with *p*-nitrophenyl decanoate (pNPC10). Moreover, the *K*m value of pNPC2 (189.73 ± 12.20 μM) was lower than that of pNPC4 (258.41 ± 5.97 μM). These results suggested that the KmYME esterase preferred short-chain pNP aliphatic ester substrates. For acyl-CoA substrates, KmYME showed a higher enzyme activity and lower *K*m value with C10-CoA (321.77 ± 34.20 nmol/min/mg and 329.20 ± 12.75 μM, respectively), compared with those of C4-CoA (48.32 ± 1.68 nmol/min/mg and 468.44 ± 20.62 μM, respectively) and succinic-CoA (44.22 ± 2.28 nmol/min/mg and 387.58 ± 3.42 μM, respectively). There was almost no enzyme activity detected with C2-CoA, indicating that KmYME preferred long-chain acyl-CoA substrates.

**TABLE 4:** Comparison of the enzymatic properties of KmYME and its mutants.

	**KmYME**		**KmYME (GYSLG→GHSMG)**		**KmYME (GYSLG→AYALA)**	
**Substrates**	**Enzyme activity (nmol/min/mg)**	***K*m (μ M)**	**Enzyme activity (nmol/min/mg)**	***K*m (μ M)**	**Enzyme activity (nmol/min/mg)**	***Km* (μ M)**
pNPC2	2303.61 ± 154.69	189.73 ± 12.20	157.80 ± 9.18	193.89 ± 10.00	N.D.	N.D.
pNPC4	600.59 ± 5.90	258.41 ± 5.97	45.67 ± 9.07	685.66 ± 34.83	N.D.	N.D.
pNPC10	N.D.	N.D.	N.D.	N.D.	N.D.	N.D.
C2-CoA	N.D.*	N.D.	N.D.	N.D.	N.D.	N.D.
C4-CoA	48.32 ± 1.68	468.44 ± 20.62	18.63 ± 0.81	1138.20 ± 185.83	N.D.	N.D.
C10-CoA	321.77 ± 34.20	329.20 ± 12.75	122.98 ± 17.86	449.88 ± 87.78	N.D.	N.D.
Succinyl-CoA	44.22 ± 2.28	387.58 ± 3.42	22.46 ± 3.22	1022.84 ± 145.83	N.D.	N.D.

N.D., not detected under standard condition.*After 3 h incubation small amount of products were detected.

Interestingly, the enzyme activities and substrate affinity of KmYME (GYSLG→GHSMG) notably declined (Table 4). However, the preference characteristics of this mutant with the pNP aliphatic esters or acyl-CoA substrates was the same as those of KmYME (Table 4). KmYME (GYSLG→AYALA) had no enzyme activities with any of the pNP aliphatic ester or acyl-CoA substrates. These results indicated that the consensus pentapeptide GXSXG was essential for the esterase and thioesterase activity of KmYME.

### Enzymatic Activity Was Required for KmYME Resistance to Inhibitors

**FIGURE 6:**
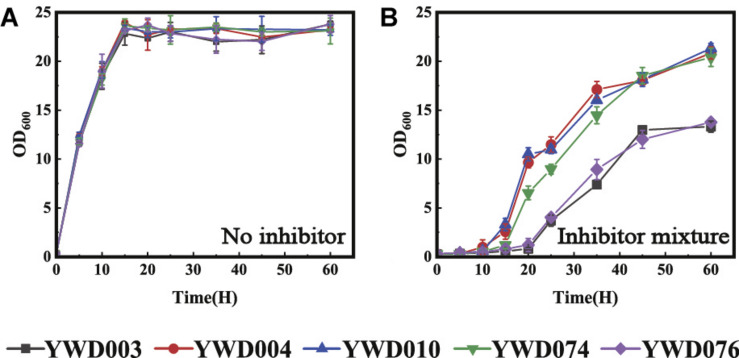
Growth of strains YWD003, YWD004, YWD010, YWD074 and YWD076 in YPD **(A)** without inhibitors, **(B)** in the presence of the inhibitor mixture. All values are the means of three biological replicates ± standard deviation.

After analysis of the KmYME esterase and thioesterase activity, their effect on the tolerance to inhibitors was evaluated by expressing KmYME or its mutants in KmYME deficient strains. The strains expressing KmYME, KmYME (GYSLG→GHSMG), or (GYSLG→AYALA) were YWD004, YWD074, and YWD076, respectively (Table 2). Then, the growth of YWD003, YWD004, YWD010, YWD074, and YWD076 was measured with or without inhibitors. As shown in Figure 6, although there was no obvious difference in the growth among the strains without inhibitors, in the presence of the inhibitor mixture (3.0 g/l acetic acid, 0.75 g/l furfural, 0.75 g/l 5-HMF, 0.3 g/l phenols), the growth of YWD076 was obviously repressed and similar to that of YWD003, and the growth of YWD074 was a little slower than that of YWD004 and YWD010. The growth performance was consistent with the enzymatic activity of KmYME and its mutants. These results suggested that the enzymatic activity (esterase or thioesterase) was necessary for the function of KmYME in the tolerance to inhibitors.

### Transcriptomic Analysis of the *KmYME* Disrupted Strain in the Presence of Multiple Inhibitors

The transcriptomic analysis of *K. marxianus* YWD001 (*KmYME* disrupted) and YWD005 (no disruption of *KmYME*) (Table 2) with or without the inhibitor mixture was conducted using RNA-sequencing (RNA-seq). The RNA-seq results were analyzed in the following two relevant pairwise comparisons of gene expression levels: YWD001-I vs. YWD001-C (*K. marxianus* YWD001 with vs. without inhibitors), and YWD005-I vs. YWD005-C (*K. marxianus* YWD005 with vs. without inhibitors).

Unexpectedly, compared to those under the no stress conditions, most of the differentially expressed genes (DEGs) (about 87.30%, data not shown) in YWD001 in the presence of multiple inhibitors (YWD001-I vs. YWD001-C) were also found in YWD005 with the same inhibitors stress conditions (YWD005-I vs. YWD005-C). Additionally, most of them showed the same up- or down-regulation trends, although the relative expression levels (the fold changes, shown as log_2_ FC) were different between the pairwise comparisons. The 215 unique DEGs (about 12.70%) (not crossed with YWD005-I vs. YWD005-C) in YWD001-I vs. YWD001-C pairwise comparison were too decentralized by KEGG or GO enrichment analysis, so we focused on the total DEGs in the YWD001-I vs. YWD001-C group, regardless of the comparison or not with those in the YWD005-I vs. YWD005-C group, especially those DEGs related to the mitochondrial respiratory chain, coenzyme-dependent proteins, NAD^+^ biosynthesis, ROS reduction, and fatty acid biosynthesis and degradation.

As shown in Additional File 2: Supplementary Table S1, in the presence of multiple inhibitors, quite a few DEGs related to NAD(P)^+^ dependence were differentially regulated. Among those DEGs related to central carbon metabolism, *ADH3/4*, *ALD2/5*, *GUT2* etc., were down-regulated and led to less NAD(P)H production. Meanwhile, all DEGs related to the tricarboxylic acid cycle (TCA cycle) such as *SDHs*, *MDH2* etc., and those related to glutamate metabolism such as *GDH1* and *UGA2*, were up-regulated. Also, *TDH2* and *ADH6* were up-regulated, suggesting an increase of NAD(P)H production. In addition, some NAD(P)H-dependent DEGs coding for dehydrogenases and oxidoreductases such as *GRE2*, *LYS1* etc., were also up-regulated in response to the resistance to the oxidative stress induced by inhibitors (Additional File 2: Supplementary Table S1). It was difficult to draw a conclusion from these transcriptome analysis results that the NAD(P)H production was enhanced in response to the stress of the inhibitors.

For those DEGs related to NAD^+^ biosynthetic enzymes and related proteins, such as *BNA3*, *FUN26*, *NMNAT*, and *URH1*, all of them were up-regulated except *PNC1*, coding for nicotinamidase (Additional File 2: Supplementary Table S1). Another gene *NUDT12*, coding for NADH pyrophosphatase, which was related to nicotinate and nicotinamide metabolism, was also up-regulated. These results suggested an enhancement of NAD^+^ production in response to the stress of multiple inhibitors.

The respiratory chain of the inner mitochondrial membrane is a unique assembly of protein complexes that transfers the electrons of reducing equivalents to molecular oxygen to generate a proton-motive force as the primary energy source for cellular ATP-synthesis (Dröse et al., 2014). For those DEGs related to the complexes within the mitochondrial respiratory chain, in both YWD005-I vs. YWD005-C and YWD001-I vs. YWD001-C pairwise comparisons, under the stress of multiple inhibitors, *NDI1* coding for rotenone-insensitive NADH-ubiquinone oxidoreductase, *NDH1* coding for the external NADH-ubiquinone oxidoreductase 1, and the *SDH*s coding for succinate dehydrogenase, and *COX2* coding for cytochrome c oxidase subunit 2 were up-regulated (Additional File 2: Supplementary Table S1). There were no significant changes in the genes encoding the F_1_F_0_ ATP synthase subunits (data not shown). These results suggested that when exposed to inhibitors, cells regulated their energy metabolism toward increased generation of ATP.

As expected, when exposed to the stress of multiple inhibitors, most of the DEGs related to ROS detoxification were up-regulated. These genes encode proteins including superoxide dismutases (*SOD1*, *SOD2*), glutathione peroxidase 2 (*GPX2*), the thioredoxin system (*TRR1, PRX1, DOT5, HYR1*) and the glutathione/glutaredoxin system (*GSH1*) etc. The only two exceptions were *CTT1* and a gene coding for glutaredoxin-like protein YLR364W, which were down-regulated (Additional File 2: Supplementary Table S1). These results indicated that the defense systems were activated to detoxify ROS and to repair the damage caused by ROS.

We also noticed that most of the DEGs related to fatty acid biosynthesis, elongation and fatty acid degradation were down-regulated except *MECR*, *PECI* and *ACADM*, coding for a probable *trans*-2-enoyl-CoA reductase, 3,2-*trans*-enoyl-CoA isomerase and acyl-CoA dehydrogenase family member 11, respectively, in both pairwise comparisons (Additional File 2: Supplementary Table S1). These data indicated that the fatty acid metabolism process was depressed by the stress of multiple inhibitors, regardless of whether *KmYME* was disrupted or not.

### Disruption of *KmYME* Reduced the Tolerance to Other Stresses in *K. marxianus*

Because the disruption of *KmYME* reduced ATP, NAD, NADP production and reduced MMP, increased ROS accumulation, and thereafter affected the tolerance to inhibitors, it is possible that the disruption of *KmYME* reduced the tolerance to other stresses. In industrial production, osmotic pressure, ethanol and temperature can affect microorganism growth and fermentation. Therefore, the cell growth of YWD003, YWD004, and YWD010 cultivated at 42°C in YPD with 180 g/L glucose, 0.5 M NaCl, or 20 g/L ethanol were conducted to evaluate the effect of the stress of sugar, salt, and ethanol, respectively. Furthermore, growth at 45°C was also conducted to evaluate the effect of temperature stress.

**FIGURE 7:**
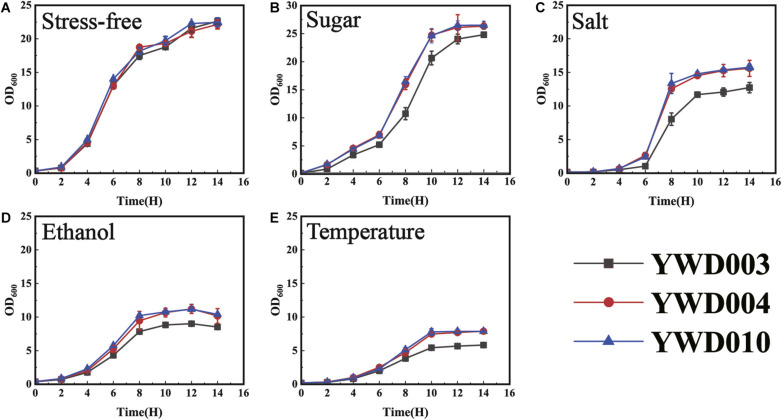
The growth of strains YWD003, YWD004, and YWD010 in YPD medium with various stresses. **(A)** No stress (42°C); **(B)** 180 g/L glucose at 42°C; **(C)** 0.5 M NaCl at 42°C; **(D)** 20 g/L ethanol at 42°C; **(E)** 45°C. All values are the means of three biological replicates ± standard deviation.

As shown in Figure 7, with the relatively high concentrations of glucose and salt (Figures 7B,C), the growth of YWD003 (*KmYME* disrupted strain) was weaker than that of strains YWD004 and YWD010, though there was no obvious difference among them without stress (Figure 7A). The YWD003 strain was also more sensitive to ethanol and weaker growth was detected with 20 g/L ethanol (Figure 7D). When the temperature was increased to 45°C, the growth of all strains decreased with a longer lag phase, lower specific growth rate, and less biomass (OD_600_) (Figure 7E); the growth of strain YWD003 showed the weakest growth among these strains. These results indicated that disruption of *KmYME* led to not only decreased tolerance to lignocellulosic inhibitors, but also decreased tolerance to osmotic pressure, ethanol, and temperature stresses.

### Double Disruption of Two Homologous Proteins Reduced the Tolerance to Inhibitors in *S. cerevisiae*

**FIGURE 8:**
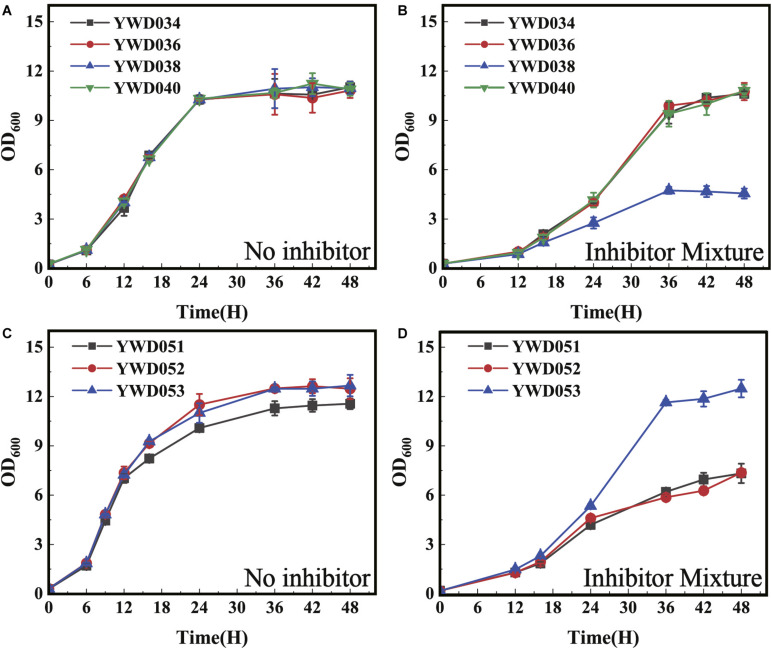
The growth of *S. cerevisiae* strains with the *KmYME* homologous genes disrupted at 30°C. **(A,C)** without inhibitors; **(B,D)** in the presence of the inhibitor mixture. All values are the means of three biological replicates ± standard deviation at each of the time points.

After confirming the decreased tolerance to the inhibitors by the disruption of *KmYME* in *K. marxianus*, we tested homologous genes in other yeast. Two ABHD-containing proteins (GenBank: NP_011545, NP_011529) homologous to KmYME were found in *S. cerevisiae*. The alignment of the amino acid sequences between KmYME and these two homologs is shown in Additional File 2: Supplementary Figure S6. Subsequently, these two genes in *S. cerevisiae* W303 1A were disrupted one by one. Two single-disruption strains (YWD034 and YWD036) and one double-disruption strain (YWD038) were obtained (Table 2). The strain YWD040 (*S. cerevisiae* W303 1A complemented with *ScURA3*) was used as the non-disrupted control. As shown in Figure 8, in the presence of the multiple inhibitor mixture, the growth of all strains decreased with a longer lag phase and YWD038 showed the worst performance (Figure 8B), though there was no obvious difference among the growth of these strains without the inhibitors (Figure 8A). These results indicated that the single disruption of *NP_011545* or *NP_011529* did not reduce the tolerance to the inhibitors, while the double disruption led *S. cerevisiae* to be more sensitive to the inhibitors.

Subsequently, the *KmYME* gene was expressed in *S. cerevisiae* YWD038 to determine if the reduced tolerance to the inhibitors could be rescued. YWD051 (YWD038 complemented with *TRP1* as a non-overexpressing control), YWD052 (*KmYME* gene expressed in YWD038), YWD053 (YWD040 complemented with the *TRP1* non-disrupted control) were obtained (Table 2) and then cultivated in YPD medium with or without inhibitors. As shown in Figures 8C,D, under the inhibitor mixture treatment, the growth of YWD051 and YWD052 was slower than that of YWD053, though there was no obvious difference among these three strains without inhibitors. This suggested that the expression of *KmYME* in YWD038 did not rescue the tolerance to inhibitors of the double-disrupted strain.

## Discussion

Exploring the toxicity of lignocellulose-derived inhibitors to yeast and developing strains with enhanced tolerance is becoming more critical in producing chemical products from lignocellulosic materials. Considering the synergistic effects of these inhibitors, the construction of a strain with tolerance to multiple inhibitors has an increased practical application value. In our previous study, the significant up-regulation of *KmYME* was detected in the presence of multiple inhibitors (Wang et al., 2018). Here, its upregulation was confirmed in the presence of multiple inhibitors or each single kind of inhibitor (Figure 1), and this uncharacterized ABHD protein aroused great interest.

*KmYME* was shown to be essential in the tolerance to inhibitors by gene disruption and retro-complementation (Figure 2). However, overexpression of *KmYME* did not enhance the tolerance (Additional File 2: Supplementary Figure S1). It is possible that the amount of KmYME required for tolerance to inhibitors was relatively small and overexpression of *KmYME* saturated that requirement. The retro-complementation with KmYME mutants with no or weak activity also suggested this possibility. Through site-directed mutagenesis of the consensus pentapeptide GXSXG, which is found in lipases/esterases and generally contains the active site serine, the low activity and no activity mutants were obtained (Table 4). The strain expressing the KmYME mutant with no activity (YWD076) did not rescue the tolerance to the inhibitors of the *KmYME* disrupted strain, while the strain expressing the low-activity mutant (YWD074) partly rescued the tolerance to the inhibitors (Figure 6). The tolerance to the inhibitors of the strains expressing the mutants was consistent with the enzymatic activity (Figure 6). ABHD11, the mammalian homolog to KmYME (Additional File 2: Supplementary Figures S6, S8), has high expression in metabolically active tissues and is related to many diseases such as Williams-Beuren syndrome (Merla et al., 2002) and lung adenocarcinoma (Wiedl et al., 2011), though the causal connection of ABHD11 to these diseases is not clear. Human ABHD11 is reported to hydrolyze pNPC2 and pNPC4, the native substrate of human ABHD11 in cells is not clear. Clearly, the identification of the function of the novel KmYME protein in the mitochondrial matrix is critical for achieving a better understanding of the ABHD family proteins. But ABHD11 cannot hydrolyze pNPC8, pNPC10 and natural lipase substrates *in vitro* (Navia-Paldanius et al., 2016). Here, KmYME also cannot hydrolyze pNPC10 *in vitro* (Table 4). It is likely that KmYME does not act as a natural lipase. Thioesterase catalyze the hydrolysis of acyl-CoAs to the free fatty acid and coenzyme A (CoASH), providing the potential to regulate intracellular CoASH pool. As products and intermediates, CoA esters (such as isovaleryl-CoA, propionyl-CoA, and methylmalonyl-CoA) was involved in fatty acid and amino acid metabolism in mitochondria. Unexpectedly, we found that the intracellular CoASH concentration of YWD003 is higher than that of YWD004 and YWD010 (Additional File 2: Supplementary Figure S9), regardless of whether inhibitor existed or not. This indicated that CoASH pool has been disturbed in *KmYME* disruption strain. The disturbance may affect the yeast cell viability with the presence of inhibitors. C2-CoA level did not changed significantly when KmYME disrupted (Additional File 2: Supplementary Figure S9). This may be due to the low activity KmYME on C2-CoA. Therefore, it is likely that disrupted *KmYME* may disturb intracellular CoASH pool and ultimately influenced ATP and NAD(P) synthesis. Though the recombinant KmYME was identified as thioesterase *in vitro*, the true substrate and catalyzed reaction remained unclear.

In addition, it is noteworthy that KmYME could hydrolyze succinyl-CoA, which is synthesized from α-Ketoglutarate and converted into succinate in TCA cycle in yeast cell. The disruption of *KmYME* may affect the succinyl-CoA metabolism, and then the energy metabolism was disturbed.

Disruption of *KmYME* interfered with NAD^+^ production. As shown in Figure 4, the presence of inhibitors led to a relatively low intracellular NAD(P) concentration, and disruption of *KmYME* caused a further decrease in intracellular NAD(P) concentrations. However, regardless of whether *KmYME* was disrupted or not, the transcriptome results indicated that genes coding for NAD^+^ biosynthetic enzymes such as *BNA3*, *FUN26*, *NMNAT*, *URH1*, and *NUDT12 etc.* were up-regulated in the presence of inhibitors (Additional File 2: Supplementary Table S1). Nevertheless, the intracellular concentration of ATP, which is the adenylyl-backbone donor for NAD^+^ (Pinson et al., 2019) decreased in the presence of inhibitors (Table 3), and this may have led to the decreased concentration of NAD pool decreased. NADP^+^ is generated from phosphorylation of NAD^+^ by NAD^+^ kinase (Love et al., 2015), so the intracellular NADP pool also decreased. In addition, it is well known that the mitochondria is a place to produce NAD(P)H through the TCA cycle and NAD(P)H is required for the conversion of furfural, HMF and phenol inhibitors into low toxicity compounds (Jönsson et al., 2013; Wang X. et al., 2016). The removal of excess ROS induced by inhibitors also requires increased NADPH (Minard and McAlister-Henn, 2001). Thus, the NAD(P)H/NAD(P)^+^ ratio was very low and showed no obvious difference among the three strains in the presence of furfural, HMF and phenols (Figure 4). Regarding the resistance to acetic acid, acetic acid is pumped out with the consumption of ATP and the procedure does not use reduced NAD(P)H co-enzyme directly (Palma et al., 2018). Furthermore, acetic acid can be converted into C2-CoA, which is a precursor substrate in the TCA, by acetyl-CoA synthetase (ACS). The reduced NAD(P)H coenzyme production may be enhanced in this situation due to the increased C2-CoA or just in response to stress, whereas the degradation of acetic acid requires less reduced NAD(P)H coenzyme. Therefore, the NAD(P)H/NAD(P)^+^ ratio in the non-disrupted strains was elevated in the presence of acetic acid.

The disruption of *KmYME* interfered with ATP production. Though the transcriptomic analysis indicated that the expression of genes related to ATP synthesis were up-regulated and the expression of genes related ATP consumption were down-regulated in the presence of inhibitors (Additional File 2: Supplementary Table S1), the intracellular ATP concentration decreased in all strains, and the *KmYME* disrupted strain (YWD003) had the lowest concentration among the strains (Table 3). A significant decrease in the ATP concentration due to the disruption of *KmYM*E indicated that KmYME affected ATP production. With the presence of oligomycin, an F_1_F_0_ ATP synthase inhibitor, the concentration of intracellular ATP decreased in a similar degree regardless of whether *KmYME* was disrupted or not (Table 3). Thus, the disruption of KmYME did not interfere in ATP synthesis directly. There may be several reasons. (1) The inhibitor leads to the disruption of the proton gradient by uncoupling the respiratory chain and the oxidative phosphorylation of ADP, so that ATP regeneration is inhibited (Jönsson et al., 2013); (2) The non-specific hydrolysis of ATP increases in the presence of furfural (Sárvári Horváth et al., 2003); (3) Yeast cells have been reported to re-direct the energy to fix the damage by reduced intracellular ATP and NAD(P)H levels either by enzymatic inhibition or consumption/regeneration of cofactors (Almeida et al., 2007; Keating et al., 2014); (4) In addition, ATP is consumed to pump out the toxic inhibitors (weak acid) from the cell (Piper et al., 1998; Sárvári Horváth et al., 2003); (5) Finally, the low concentration of NADH could lead to decreased ATP concentration. Therefore, it is not unexpected that the intracellular concentration of ATP decreased in the presence of inhibitors (Table 3). In our study, the reduced intracellular concentration of NAD(P)H in KmYME disrupted strains compared to non-disrupted strains (Figure 4) led to a more severe decrease in ATP production (Table 3).

Disruption of KmYME led to a higher accumulation of ROS. Acetic acid, furfural and phenols have been reported to induce ROS accumulation (Ludovico et al., 2001; Allen et al., 2010; Fletcher et al., 2019) in yeast cells and excessive ROS can damage many cellular components, including DNA, proteins and lipids (Jamieson, 1998) and ultimately lead to apoptosis of the cell (Farrugia and Balzan, 2012). In our study, intracellular ROS level increased in the presence of inhibitors. Furthermore, the *KmYME* disrupted strain (YWD003) showed more ROS accumulation (Figure 5D). The RNA-seq results showed that regardless of whether *KmYME* was disrupted or not, genes coding for proteins responsible for ROS removal and maintaining the redox balance such as superoxide dismutases, peroxidase, thioredoxin reductase, glutathione/glutaredoxin systems were up-regulated in response to the stress of multiple inhibitors (Additional File 2: Supplementary Table S1). In addition, KmYME was shown to have no peroxidase activity. These results hinted that the increased accumulation of ROS in the *KmYME* disrupted strain was not due to the change in expression levels of ROS removal enzymes. Therefore, disruption of *KmYME* led to increased ROS accumulation due to the decreased reducing power of NAD(P)H, which is used by thioredoxin peroxidase and glutathione oxioreductase (Minard and McAlister-Henn, 2001). Interestingly, the amount of cell death was not similar to ROS levels (Figure 5D); this may be explained by other lethal effects of the inhibitors, in addition to ROS accumulation.

For the KmYME disruption strain, because the expression pattern of the NAD(P)H dependent genes in the *KmYME* disrupted strain and non-disrupted strains were similar, the decreased NAD(P)H/NAD(P)^+^ ratio was not due to the change in gene expression, and may be due to the interference of the KmYME disruption in NAD (NAD^+^, NADH) and NADP (NADP^+^, NADPH) synthesis, especially reductive NAD(P)H synthesis. Here, we proposed a hypothesis on the resistance mechanism of KmYME. Disrupted KmYME may affect intracellular CoASH pool to induce metabolic flux redistribution and then the synthetic of ATP and NAD(P), especially reductive NAD(P)H is decreased in the presence of inhibitors. In previous studies, ATP and NAD(P)H are necessary for the detoxification of weak acids, furan derivatives and phenolic inhibitors (Keating et al., 2014; Wang S. et al., 2016). NADPH donates electrons eliminate ROS generation (Minard and McAlister-Henn, 2001). Moreover, ATP and NAD(P)H are necessary for biomolecule synthesis and cell division. The detoxification of inhibitors consumed a large amount of ATP and NAD(P)H. As a result, the intracellular ATP and NAD(P)H concentrations decreased in the presence of inhibitors (Table 3 and Figure 4). The disruption of *KmYME* further reduced the ability to synthesize ATP and NAD(P)H; therefore, the shortage of ATP and NAD(P)H caused mitochondrial dysfunction, which was characterized by lower MMP and increased ROS accumulation and eventually led the cell to be sensitive to the inhibitors. This may also be the reason why disruption of *KmYME* reduced the tolerance of cells to other stresses including ethanol, temperature, and osmotic pressure (Figure 7). However, the native substrate and specific mechanisms of KmYME is unknown.

The KmYME disrupted strain had no obvious changes in death rate and growth compared to non-disrupted strains in the presence of the phenolic mixture. Because phenolic compounds cause the loss of membrane integrity, including the cell membrane and mitochondrial membranes, they inhibit microbial growth and fermentation (Palmqvist and Hahn-Hägerdal, 2000; Almeida et al., 2007). Because of the complexity of the compound structure with different functional groups and functional side groups, the mechanism of yeast tolerance to each phenolic compound is quite different (Adeboye et al., 2014). In this study, KmYME may not contribute to membrane repair. Also, the contribution of KmYME to the tolerance of phenolic inhibitors may be eclipsed due to the different tolerance mechanisms to the phenolic compounds in the mixture.

Two homologous genes were found in the genome of *S. cerevisiae* (Additional File 2: Supplementary Figures S6, S8), with no report on the determination of function. In our study, the disruption of either *NP_011545* (YGR031W) or *NP_011529* in *S. cerevisiae* did not change the tolerance to the inhibitors (Figure 8). However, the double disruption led to a decreased tolerance to the inhibitors (Figure 8). We speculated that the function of NP_011545 or NP_011529 may be similar or overlapping. However, overexpression of *KmYME* could not remedy the decreased tolerance to the inhibitors of the double disruption strain of *S. cerevisiae*, suggesting that the mechanisms of KmYME in tolerance to inhibitors in *K. marxianus* may be different than the homologous proteins in *S. cerevisiae*. However, this novel ABHD family protein located in the mitochondrial matrix was applicable to other stresses and other organisms, which suggests a wide variety of promising applications.

## Conclusion

An uncharacterized ABHD protein KmYME located in mitochondrial matrix and the esterase/thioesterase enzymatic activity was required for tolerance to multiple inhibitors. Tolerance to the inhibitors decreased in the *KmYME* disrupted strain while overexpression of *KmYME* did not improve the tolerance to inhibitors in *K. marxianus*. Disruption of K*mYME* did not result in a significant change of gene expression at the transcriptional level; thus, it was possible that KmYME affected the tolerance to the inhibitors through interfering with intracellular CoASH pool to reduce NAD(P)^+^, NAD(P)H and/or ATP synthesis. However, the specific enzyme substrates of KmYME is unknown, it remains to be further study. Disruption of two possible homologous genes in *S. cerevisiae* also reduced tolerance to inhibitors. The results of this study will enhance the understanding of the tolerance mechanisms in yeast to lignocellulosic hydrolysate inhibitors.

## Data Availability Statement

The datasets presented in this study can be found in online repositories. The names of the repositories and accession numbers can be found in the article/Supplementary Material.

## Author Contributions

DaW designed the research, performed the most of the experiments, and collected the data. DoW carried out the RT-PCR experiments and conducted the analyses of transcriptomic. JH and DoW conceived the concept and design of the experiment. All authors contributed to writing the manuscript, read and approved the final manuscript.

## Conflict of Interest

The authors declare that the research was conducted in the absence of any commercial or financial relationships that could be construed as a potential conflict of interest.

## Footnotes

1 http://tophat.cbcb.umd.edu

## Abbreviations

C2-CoA: acetyl-CoA
C4-CoA: butyryl-CoA
C10-CoA: decanoyl-CoA
DCW: dry cell weight
DEGs: differentially expressed genes
DTNB: 5,5’-Dithiobis-(2-nitrobenzoic acid)
Log_2_ FC: log_2_ transformed fold change values
MMP: mitochondrial membrane potential
PBS: phosphate buffer saline
PI: propidium iodide
pNPC10: *p*-nitrophenyl decanoate
pNPC2: *p*-nitrophenyl acetate
pNPC4: *p*-nitrophenyl butyrate
Rh123: rhodamine 123
ROS: reactive oxygen species
SD: synthetic dropout medium
TCA cycle: tricarboxylic acid cycle.
