# Supplementary material for: Effect of a Novel Alpha/Beta Hydrolase Domain Protein on Tolerance of K. marxianus to Lignocellulosic Biomass Derived Inhibitors
Source: Front Bioeng Biotechnol. 2020 Jul 24;8:844. doi: 10.3389/fbioe.2020.00844 (PMC7396682; doi:10.3389/fbioe.2020.00844)
Supplement: Supplementary file 1 [file Data_Sheet_1.pdf]

Additional file 1

Plasmid construction

The plasmid for *KmYME* disruption cassette was constructed as following. *KmYME* Open reading frame (ORF) was amplified from the genomic DNA of *K. marxianus* NBRC1777 with primer pairs KmYME-F and KmYME-R (Additional file 1: Table S1) and inserted into pGEM-T easy (Promega Corporation, Madison, WI, USA) to obtain pWD001. Then, the resultant plasmid pWD001 was linearized to remove a 120 bp sequence of *KmYME* ORF by PCR using primer pairs KmYME-F2 and KmYME-R2 (Additional file 1: Table S1, Fig. S1). A *ScURA3* gene expression cassette was amplified with primer pairs ScURA3-SMAI-FULL-F and ScURA3-SMAI-FULL-R (Additional file 1: Table S1) using genomic DNA of *S. cerevisiae* W303 1A as temperate. After the ScURA3 fragment was digested by *Sma*I, it was inserted into linearized pWD001 to obtain plasmid pWD002 in which part of KmYME ORF was substituted by *ScURA3* expression cassette. pWD002 was used to amplify the disruption cassette of *KmYME* (Additional file 1: Fig. S1). The plasmid pWD024 and pWD025 for disruption cassettes of *KmYME* homologous genes in *S. cerevisiae* (*NP_011545* and *NP_011529*) were constructed similar as pWD002.

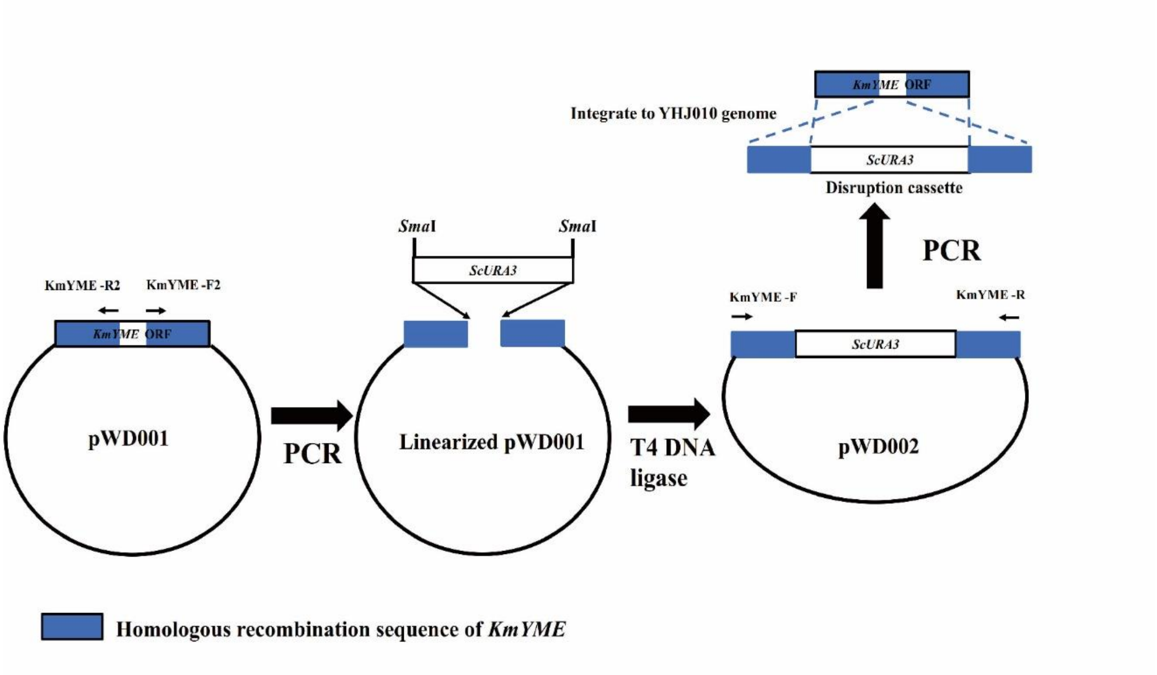

Fig. S1 Construction of disruption cassette of *KmYME*. The plasmid pWD001 containing *KmYME* with part of ORF frame removed was amplified with PCR from pWD001 and ligated with a *ScURA3* expression cassette. The resultant plasmid pWD002 was used to amplify the *KmYME* disruption cassette.

The plasmid for *KmYME* overexpression was constructed as following. Full length DNA of *KmYME* ORF was amplified from pWD001 using the primer pairs KmYME-EcoRI-F and KmYME-NotI-R (Additional file 1: Table S1). Then the ORF was inserted into plasmid YEGAP (Hong et al., 2001) between the *Eco*RI and *Not*I sites to obtain the plasmid pWD003 which was used to overexpress *KmYME* in *K. marxianus* YHJ010.

The plasmids of fluorescence protein fusion for intracellular location were constructed as following. The expression cassettes of KmYME-EGFP and KmCox-RFP were obtained by fusing *KmYME* with *EGFP* and fusing *KmCox* with *RFP* respectively. *KmYME* ORF without stop codon was obtained from pWD001 using primer pairs KmYME-EcoRI-F and KmYME-fusion-R. *EGFP* ORF was obtained from pCG (Hong et al., 2008) using primer pairs EGFP-fusion-F and EGFP-NotI-R. Then KmYME-EGFP fused fragment was obtained by overlap extension PCR with KmYME-EcoRI-F and EGFP-NotI-R primers pair. *KmCox* ORF without stop codon was amplified from the genomic DNA of *K. marxianus* NBRC1777 with primer pair KmCox-EcoRI-F and KmCox-fusion-R. *RFP* ORF amplified from pCF.1676 (Li et al., 2015) with RFP-fusion-F and RFP-NotI-R primer pairs. Subsequently, *KmCox-RFP* fused fragment was obtained through overlap extension PCR with KmCox-EcoRI-F and RFP-NotI-R as primers pair. Then *KmYME-EGFP* fragment or *KmCox-RFP* fragment was digested by *Eco*RI and *Not*I and then inserted into YEUKmPGK (Yang et al., 2015) or YEGAP between the *Eco*RI and *Not*I sites to obtain the plasmid pWD005 and pWD006, respectively. pWD005 and pWD006 were used to expression fusion protein in *K. marxianus* to locate KmYME. After digested by *Eco*RI and *Not*I, *EGFP* ORF was inserted into YEUKmPGK between the *Eco*RI and *Not*I sites to obtain the plasmid pWD004 which was used to express EGFP. *KmCox-RFP* fragment was digested by *Eco*RI and *Not*I and then inserted into YEUKmPGK to obtain the plasmid pWD026 which was used to express Cox-RFP with URA3 selection marker to observe mitochondrial morphology.

The plasmids used to determine the mitochondrial signal peptide of KmYME were constructed as following. The plasmids for expression truncated KmYME (1-20 aa)-EGFP and KmYME (1-40 aa)-EGFP were constructed with the frame of plasmid pWD004 using primer pairs EGFP-F3 and KmYME-R3, EGFP-F4 and KmYME-R4, respectively. Then the linearized plasmids were ligated using Exnase II (Vazyme Biotech Co., Ltd, Nanjing, China). The resultant plasmids were named pWD007 and pWD008 (Additional file 1: Fig. S2). Likewise, KmYME (41-360 aa)-EGFP fragment was amplified from pWD004 with KmYME-EcoRI-F2 and EGFP-NotI-R, then digested by *Eco*RI and *Not*I and inserted into YEUKmPGK between the *Eco*RI and *Not*I sites to obtain the plasmid pWD009.

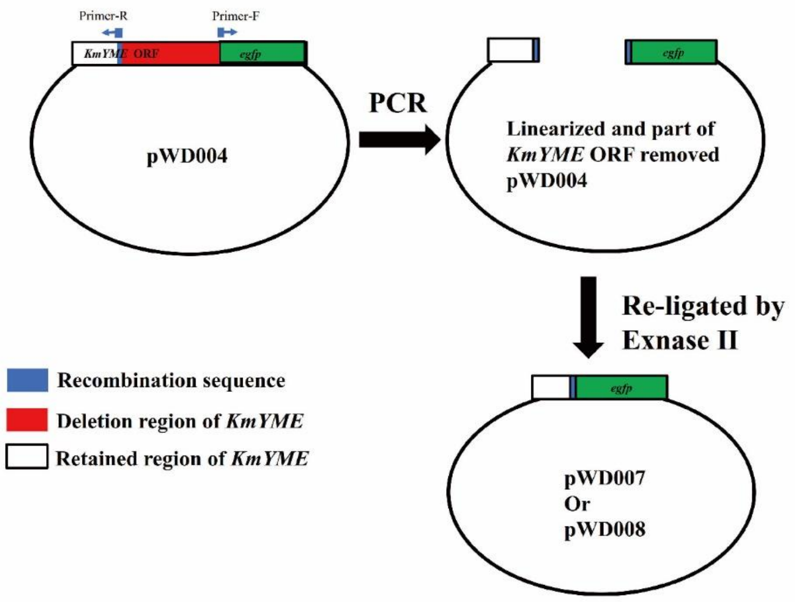

Fig. S2 Construction of the plasmid for KmYME mitochondrial signal peptide determination.

The plasmid used to express KmYME in *E. coli* was constructed as following. KmYME ORF was amplified from pWD001 with KmYME-NdeI-F and KmYME-XhoI-R. The fragment was inserted into *Nde*I and *Xho*I digested pET22b to obtain the plasmid pWD010.

The plasmids for expression of KmYME mutants were constructed through site-directed mutagenesis of GXSXG motif as following. The amino acid residues GYSLG in KmYME were replaced with GHSMG or AYALA using PCR-based amplification of the entire pWD003 and pWD010 construct. The primer pairs KmYME-H-F and KmYME-H-R were used to change GYSLG into GHSMG. And the primer pairs KmYME-A-F and KmYME-A-R were used to change GYSLG mutant into AYALA. Then the fragments were ligated using Exnase II. The plasmids containing the mutant of KmYME were named as pWD032 (GYSLG→GHSMG in pWD003), pWD033 (GYSLG→AYALA in pWD003), pWD034 (GYSLG→GHSMG in pWD010), pWD035 (GYSLG→AYALA in pWD010).

Strains construction

KmYME disrupted strain was constructed as following. The disruption cassette of *KmYME* was amplified with primers KmYME -F/R (Additional file 1: Table. S1) from plasmid pWD002 and then transformed into YHJ010. The transformants were screened on SD medium without Uracil. The obtained strain in which *KmYME* was disrupted was named YWD001 (Additional file 1: Fig. S3). The *ScURA3* expression cassette, which was the selection marker for disruption cassette, was transformed into YHJ010 to obtain the non-disruption control strain YWD005 (Additional file 1: Fig. S3)

In order to overexpress *KmYME*, linearized pWD003 was transformed into YHJ010

and screened on SD medium without tryptophan and *KmYME* overexpression strain YWD002 was obtained (Additional file 1: Fig. S3). The *TRP1* expression cassette was then transformed into YHJ010 to construct non- overexpression strain YWD009 (Additional file 1: Fig. S3). pWD003 was linearized and transformed into strain YWD001 to construct *KmYME* retro-complementation strain YWD004 (Additional file 1: Fig. S3). pWD032 and pWD033 which contained the mutants of *KmYME* were also transformed into YWD001 to obtain YWD074 and YWD076, respectively. Empty plasmid YEGAP (Hong et al., 2001) was linearized and transformed into YWD001 and YWD005 to obtain YWD003 and YWD010, respectively.

To determine the location of KmYME, the strains co-expression of EGFP and KmCox-RFP (control strain) or co-expression of KmYME-EGFP and KmCox-RFP were constructed. Specifically, *K. marxianus* YHJ010 was transformed with linearized plasmid pWD004 and pWD006 to obtain strain YWD024 in which EGFP and KmCox-RFP were co-expressed as a control. On the other hand, *K. marxianus* YHJ010 was subsequently transformed with linearized pWD005 and pWD006 to obtain strain YWD026 (Additional file 1: Fig. S3) in which KmYME-EGFP and KmCox-RFP were co-expressed to determine KmYME intracellular location.

To determine the mitochondrial signal peptide of KmYME, the strains expressing various truncated KmYME fused with EGFP were constructed. *K. marxianus* YHJ010 was transformed with plasmid pWD004, pWD005, pWD007, pWD008 or pWD009 to obtain YWD021, YWD022, YWD028, YWD030, or YWD032, corresponding to the expression of EGFP, KmYME (full length)-EGFP, KmYME (1-20 aa)-EGFP, KmYME (1-40 aa)-EGFP, and KmYME (41-360 aa)-EGFP, respectively (Additional file 1: Fig. S3).

To observe the mitochondrial morphological, the strains expressing KmCox-RFP were constructed to assist the mitochondria detection. A *ScURA3* disruption cassette was amplified from pMD18T-ΔScURA3 (Zhang et al., 2016) and transformed into YWD003, YWD004, and YWD010 to disrupt *ScURA3* and selected on an SD plate containing uracil and 0.1% 5'-fluoro-orotic acid (5'-FOA). Next, the obtained positive strains were transformed with linearized pWD026 to obtain YWD046, YWD047, and YWD048, respectively. YWD046, YWD047, and YWD048 expressed KmCox-RFP in *KmYME* disrupted strain, *KmYME* retro-complement stain and *KmYME* non-disruption stain (wild type control), respectively (Additional file 1: Fig. S3).

The homologous genes of *KmYME* in *S. cerevisiae* were also disrupted. The gene *NP_011545* or *NP_011529* disruption cassettes were amplified with primer pairs NP_011545-F/R or NP_011529-F/R (Additional file 1: Table. S1) from plasmid pWD024 or pWD025 and then transformed into *S. cerevisiae* W303 1A. Strains YWD034 and YWD036 were obtained (Additional file 1: Fig. S3). To obtain double disruption strain, the *ScURA3* in YWD034 was disrupted through transformed by a *ScURA3* disruption cassette which was amplified from pMD18T-ΔScURA3. The *ScURA3* re-disrupted strain was selected on an SD plate containing uracil and 0.1% 5'-fluoro-orotic acid (5'-FOA). Then the obtained strain YWD037 was transformed by *NP_011529* disruption cassette to obtain double disruption stain YWD038. The non-disruption control strain YWD040 was obtained through transformation of *S.*

*cerevisia*e W303 1A with *ScURA3* expression cassette (Additional file 1: Fig. S3). To evaluate if KmYME could rescue the decreased inhibitors tolerance of double disruption strain of *S. cerevisiae*. Linearized YEGAP or pWD003 was transformed into YWD038 and screened on SD plate supplied leucine, adenylate, and histidine to obtain YWD051 (as non-overexpression control) or YWD052 (KmYME overexpression), respectively. Then, linearized YEGAP was transformed into YWD040 and YWD053 was obtained (as non-disruption control) (Additional file 1: Fig. S3).

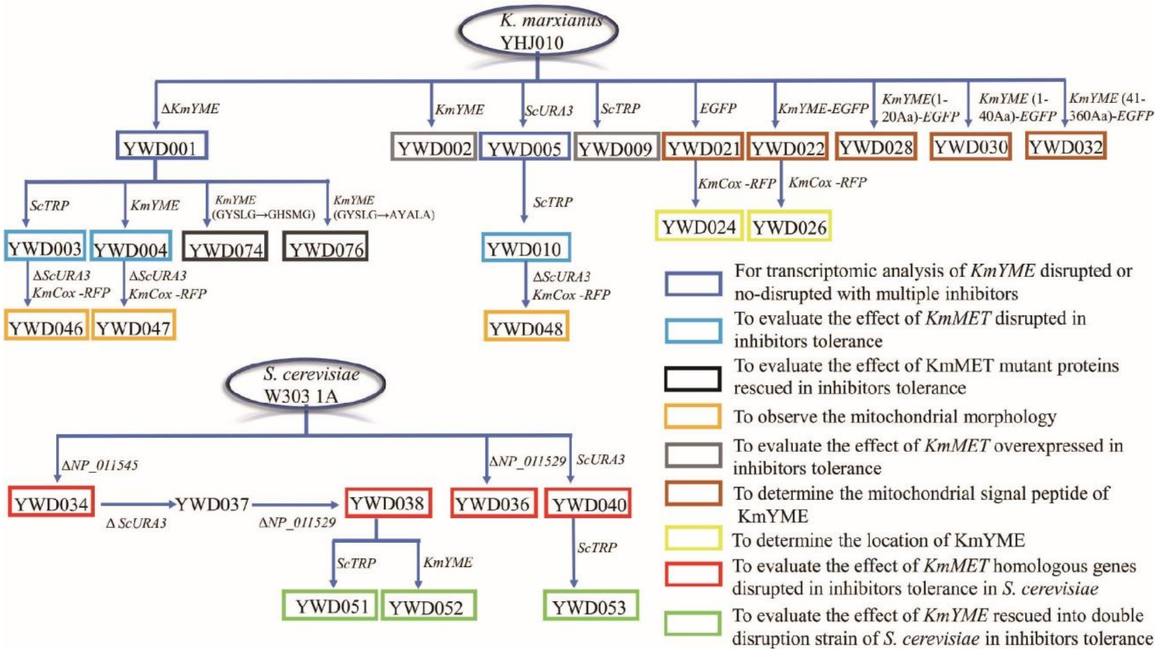

Fig. S3 Schematic diagram of the strains construction. A key for the colored boxes with strains is shown in the lower right corner.

Table S1 Primers used in this study

**Primers**	**sequence**[a]
KmYME-F	5’-ATGCTTCTCGCTTACACCGTCAG-3’
KmYME-R	5’-TTAATCTCTAGCACTTTTGAGAG-3’
KmYME-EcoRI-F	5’-AAA***gaattc***ATGCTTCTCGCTTACACCGTCAG-3’
KmYME-NotI-R	5’-ATA***gcggccgc***TTAATCTCTAGCACTTTTGAGAG-3’
KmYME-F2	5’-CAAGCAGCACTGAAACGTTCTGG-3’
KmYME-R2	5’-GACACAAAGCCTCGGAGGCATT-3’
ScURA3-SMAI-FULL-F	5′ -TCC***cccggg***TATTTAGAAAAATAAACAAATAG-3′
ScURA3-SMAI-FULL-R	5′ - TCC***cccggg***AATGCGTACTTATATGCGTC-3′
KmYME-fusion-R	5’-GCTCACACTACCACTACCTGAACCATCTCTAGCACTTTTGAGAGAT-3’
EGFP-fusion-F	5’AGAGATGGTTCAGGTAGTGGTAGTGTGAGCAAGGGCGAGGAGCTGTT-3’
EGFP-NotI-R	5’-TTA***gcggccgc***TTACTTGTACAGCTCGTCCATGCC-3’
EGFP-EcoRI-F	5’-GTA***gaattc***ATGGTGAGCAAGGGCGAGGAGCTG-3’
KmCox-EcoRI-F	5'-CTA***gaattc***ATGTTGTCTCTACGTCAATCCCTAT-3'

KmCox-fusion-R	5'-ACTACCACTACCTGAACCGTGGTGACCTTCTTGAGGAACACCC-3'
RFP-fusion-F	5'-CACGGTTCAGGTAGTGGTAGTGTGTCTAAGGGCGAAGAGCTGAT-3'
RFP-NotI-R	5'-CTT***<u>gcggccgc</u>***TCAATTAAGTTTGTGCCCCAGTT-3'
KmYME-R3	5'-GCATACTCTGGTTCAGGTAGTGGTAGTGTG-3'
EGFP-F3	5'-TACCTGAACCAGAGTATGCTCTTCTGGTAAAAG-3'
KmYME-R4	5'-CAAGGAAACTGGTTCAGGTAGTGGTAGTGTGAG-3'
EGFP-F4	5'-TACCTGAACCAGTTTCCTTGATTGGCAATGGC-3'
KmYME-EcoRI-F2	5'CTA***<u>gaattc</u>***ATGGTCGATATGGCATATGATCTG3'
KmYME-NdeI-F	5'-AGGAGATATA***<u>catatg</u>***CTTCTCGCTTACACCGTC-3'
KmYME-XhoI-R	5'-GTGGTGGTG***<u>ctcgag</u>***ATCTCTAGCACTTTTGAGAG-3'
NP_011545-F	5'-CATCAGCAAGTGTCCGATGCCTTCTC-3'
NP_011545-F2	5'-TTAAAGGCAAGCGCCCCGGAGCAAG-3'
NP_011545-R	5'-TGACGATCTTATCGTATGCATCAGC-3'
NP_011545-R2	5'-CATTGTACCGTTACCAATTCTCCTC-3'
NP_011529-F	5'-GATCAGATTCACCGTCATGAGTGTAG-3'
NP_011529-F2	5'-GATTTTGAATAAAATCTTGATGTACG-3'
NP_011529-R	5'-TATGTATAGTTCTCCTTCAAGGAGAC-3'
NP_011529-R2	5'-CATTTGTAAGCCCTCTTCCCACGG-3'
KmYME-H-F	5'-CCTTGTGGGTCACTCTATGGGTGCTAAGGTTGCCATGCTTG-3'
KmYME-H-R	5'-CCTTAGCACCCATAGAGTGACCCACAAGGTTAACCTTACCC-3'
KmYME-A-F	5'-CTTGTGGCATATGCTTTGGCAGCTAAGGTTGCCATGCTTGCCTGTT -3'
KmYME-A-R	5'-CTTAGCTGCCAAAGCATATGCCACAAGGTTAACCTTACCCAATG -3'
KmYME-RT-F	5' CCCTTGTGAAATCTTGCGTTAAGTTG3'
KmYME-RT-R	5' AATGTAGCATACCGTCGTCATAGTTG 3'
ACT1-RT-F	5'TTGGCTGGTAGAGACATCACTGAC3'
ACT1-RT-R	5' AGCAGATGATTGAGAAGCGGTTTG 3'

[a] The restriction enzyme site is bolded, italicized and underlined.

References

Hong, J., Tamaki, H., Akiba, S., Yamamoto, K., & Kumagai, H. (2001). Cloning of a gene encoding a highly stable endo-β-1,4-glucanase from *Aspergillus niger* and its expression in yeast. *Journal of Bioscience and Bioengineering, 92*(5), 434-441. doi:https://doi.org/10.1016/S1389-1723(01)80292-9

Hong, J., Ye, X., Wang, Y., & Zhang, Y. H. P. (2008). Bioseparation of recombinant cellulose-binding module-proteins by affinity adsorption on an ultra-high-capacity cellulosic adsorbent. *Analytica Chimica Acta, 621*(2), 193-199. doi:https://doi.org/10.1016/j.aca.2008.05.041

Li, T., Zheng, F., Cheung, M., Wang, F., & Fu, C. (2015). Fission yeast mitochondria are distributed by dynamic microtubules in a motor-independent manner. *Scientific Reports, 5*, 11023. doi:https://doi.org/10.1038/srep11023

Yang, C., Hu, S., Zhu, S., Wang, D., Gao, X., & Hong, J. (2015). Characterizing yeast promoters used in *Kluyveromyces marxianus*. *World Journal of Microbiology and Biotechnology, 31*(10), 1641-1646.

doi:https://doi.org/10.1007/s11274-015-1899-x

Zhang, B., Zhang, J., Wang, D., Han, R., Ding, R., Gao, X., Sun, L., & Hong, J. (2016). Simultaneous fermentation of glucose and xylose at elevated temperatures co-produces ethanol and xylitol through overexpression of a xylose-specific transporter in engineered *Kluyveromyces marxianus*. *Bioresource Technology, 216*, 227-237. doi:https://doi.org/10.1016/j.biortech.2016.05.068
