# Supplementary material for: Effect of a Novel Alpha/Beta Hydrolase Domain Protein on Tolerance of K. marxianus to Lignocellulosic Biomass Derived Inhibitors
Source: Front Bioeng Biotechnol. 2020 Jul 24;8:844. doi: 10.3389/fbioe.2020.00844 (PMC7396682; doi:10.3389/fbioe.2020.00844)
Supplement: Supplementary file 2 [file Data_Sheet_2.pdf]

Additional file 2

Table S1 Functional categories and fold changes of comparative expression[a] (in form of log_2FC) of pairwise comparisons (YWD001-I vs YWD001-C and YWD005-I vs YWD005-C) of differentially expressed genes in tolerance to multiple inhibitors.

Seq ID	*Gene*	Description	YWD001-C fpkm	YWD001-I fpkm	log_2FC (YWD001-I/YWD001-C)	YWD005-C fpkm	YWD005-I fpkm	log_2FC (YWD005-I/YWD005-C)
**DEGs related to NADH and NADPH-dependent proteins**								
Central carbon metabolism								
KMAR_10659	*XR*	NADPH-dependent D-xylose reductase	782.13	154.77	-1.98	738.84	139.21	-1.90
KMAR_20285	*TDH2*	glyceraldehyde-3-phosphate dehydrogenase 2	5.51	16.49	1.94	4.48	19.32	2.62
KMAR_80296	*ADH3*	alcohol dehydrogenase III	31.75	6.06	-2.03	17.78	5.49	-1.18
KMAR_20152	*ADH4*	alcohol dehydrogenase 4	318.33	31.44	-2.98	452.78	39.68	-3.00
KMAR_80326	*ADH6*	NADP-dependent alcohol dehydrogenase 6	193.87	6505.20	5.43	90.06	6699.52	6.73
KMAR_20637	*ALD2*	aldehyde dehydrogenase [NAD(P)+]	35.58	10.33	-1.42	29.02	11.69	-0.79
KMAR_40408	*ALD5*	aldehyde dehydrogenase 5	58.30	16.84	-1.43	103.45	14.74	-2.29
KMAR_30696	*GUT2*	glycerol-3-phosphate dehydrogenase	16.40	4.84	-1.39	17.92	4.85	-1.36
KMAR_60328	*MAE1*	NAD-dependent malic enzyme	132.41	487.64	2.25	173.16	552.54	2.20
KMAR_60167	*MDH2*	malate dehydrogenase	1557.56	2456.60	1.01	1184.36	2224.55	1.42
KMAR_20443	*SDH1*	succinate dehydrogenase [ubiquinone]	531.49	1410.57	1.78	416.82	1422.53	2.28
KMAR_30112	*SDH3*	succinate dehydrogenase [ubiquinone]	790.26	1486.46	1.23	532.50	1396.27	1.86
KMAR_50521	*SDH4*	succinate dehydrogenase [ubiquinone]	275.12	767.50	1.79	209.08	735.92	2.27

Glutamate metabolism								
KMAR_50015	*GDH1*	NADP-specific glutamate dehydrogenase 2	410.62	1613.04	2.34	316.63	1232.59	2.48
KMAR_20698	*GDH2*	NAD-specific glutamate dehydrogenase	73.48	15.77	-1.85	84.66	16.75	-1.81
KMAR_60127	*UGA2*	succinate-semialdehyde dehydrogenase [NADP+]	176.66	276.00	1.01	94.80	253.94	1.94
Others								
KMAR_40373	*LYS1*	saccharopine dehydrogenase [NAD+]	373.81	764.98	1.39	279.02	692.76	1.83
KMAR_10058		putative NADPH dehydrogenase C23G7.10c	193.38	396.04	1.40	180.40	415.30	1.72
KMAR_30702		putative uncharacterized oxidoreductase YGL039W	91.01	326.66	2.09	51.11	258.29	2.61
KMAR_40624	*GRE2*	NADPH-dependent methylglyoxal reductase GRE2	593.39	4682.50	3.34	367.83	3905.15	3.92
KMAR_50448		sulfite reductase [NADPH]	109.90	170.95	1.01	51.34	141.10	1.99
**NAD+ biosynthetic enzymes and nicotinate metabolism**								
KMAR_50064	*BNA3*	probable kynurenine--oxoglutarate transaminase BNA3	269.04	642.30	1.61	109.52	499.84	2.70
KMAR_30218	*FUN26*	nucleoside transporter FUN26	36.89	96.93	1.76	34.72	92.71	1.94
KMAR_40275	*PNC1*	nicotinamidase	196.41	62.21	-1.32	177.70	59.02	-1.11
KMAR_40052	*NMNAT*	nicotinamide-nucleotide adenylyltransferase 1	202.07	949.06	2.60	183.83	935.53	2.87
KMAR_10267	*URH1*	uridine nucleosidase	30.29	144.65	2.61	24.28	171.93	3.33
KMAR_10620	*NUDT12*	NADH pyrophosphatase	67.79	237.99	2.17	40.19	231.27	3.04
**Mitochondrial respiratory chain**								
NADH dehydrogenase								

KMAR_10252	*NDI1*	rotenone-insensitive NADH-ubiquinone oxidoreductase	116.60	232.84	1.36	108.36	251.74	1.74
KMAR_10671	*NDH1*	external NADH-ubiquinone oxidoreductase 1	263.79	1011.58	2.33	220.59	1029.66	2.75
Succinate dehydrogenase								
KMAR_20443	*SDH1*	succinate dehydrogenase [ubiquinone]	531.49	1410.57	1.78	416.82	1422.53	2.28
KMAR_30112	*SDH3*	succinate dehydrogenase [ubiquinone]	790.26	1486.46	1.23	532.50	1396.27	1.86
KMAR_50521	*SDH4*	succinate dehydrogenase [ubiquinone]	275.12	767.50	1.79	209.08	735.92	2.27
Cytochrome c oxidase								
KMAR_90002	*COX2*	cytochrome c oxidase subunit 2	0.56	1.92	2.12	0.58	2.00	2.26
**Fatty acid biosynthesis and elongation**								
KMAR_30147	*PHS1*	protein PHS1	143.88	34.60	-1.72	176.07	32.88	-1.94
KMAR_10176	*TSC13*	enoyl reductase TSC13	216.10	67.10	-1.33	235.58	66.32	-1.32
KMAR_10140	*ELO2*	elongation of fatty acids protein 2	215.23	53.86	-1.64	281.91	52.29	-1.92
KMAR_60262	*MECR*	probable trans-2-enoyl-CoA reductase	573.72	1791.16	2.00	391.72	1667.72	2.60
KMAR_60084	*ACOT1_2_4*	peroxisomal acyl-coenzyme A thioester hydrolase 1	151.00	48.24	-1.29	130.94	47.98	-0.94
KMAR_10220	*OLE1*	acyl-CoA desaturase 1	3158.93	1105.93	-1.15	4277.64	1112.68	-1.42
KMAR_40523	*FAS1*	fatty acid synthase subunit beta	83.97	12.21	-2.32	104.78	14.28	-2.27
KMAR_70200	*FAS2*	fatty acid synthase subunit alpha	370.25	114.02	-1.32	488.22	119.96	-1.49
**Fatty acid degradation**								
KMAR_10192	*PECI*	3,2-trans-enoyl-CoA	12.10	21.73	1.20	7.78	21.43	1.96

		isomerase						
KMAR_60375	*ERG10*	acetyl-CoA acetyltransferase	410.09	157.75	-1.02	600.35	148.05	-1.50
KMAR_60033	*ACADM, ACD*	acyl-CoA dehydrogenase family member 11	1.97	37.84	4.63	0.85	38.62	6.03
KMAR_80296	*ADH3*	alcohol dehydrogenase III	31.75	6.06	-2.03	17.78	5.49	-1.18
KMAR_20152	*ADH4*	alcohol dehydrogenase 4	318.33	31.44	-2.98	452.78	39.68	-3.00
KMAR_40408	*ALD5*	aldehyde dehydrogenase 5	58.30	16.84	-1.43	103.45	14.74	-2.29
**redox related protein**								
KMAR_70075	*SOD1*	Cu/Zn superoxidedismutase	370.40	4151.37	3.79	252.75	3266.87	4.13
KMAR_20527	*SOD2*	superoxide dismutase [Mn]	490.64	966.51	1.32	334.13	835.44	1.81
KMAR_40317		superoxide dismutase 1 copper chaperone	302.10	724.70	1.61	259.72	699.16	1.92
KMAR_50400	*CTT1*	catalase T	1953.39	577.94	-1.39	1850.05	510.75	-1.33
KMAR_10658	*TRR1*	thioredoxin reductase	1490.87	2419.97	1.06	1291.45	2204.93	1.28
KMAR_80342	*PRX1*	mitochondrial peroxiredoxin PRX1	775.67	4239.09	2.80	334.53	3899.19	4.04
KMAR_20388	*HYR1*	peroxiredoxin HYR1	261.11	717.38	1.79	306.75	615.62	1.48
KMAR_50387	*DOT5*	peroxiredoxin DOT5	525.71	87.35	-2.25	543.16	84.97	-2.20
KMAR_10650	*GPX2*	glutathione peroxidase 2	43.20	129.86	1.90	46.57	149.99	2.14
KMAR_30143	*GSH1*	glutamate--cysteine ligase	352.51	696.73	1.35	287.61	624.80	1.65
KMAR_30340	*GRX5*	monothiol glutaredoxin-5	368.42	1280.56	2.16	309.69	1256.28	2.52

[a] Only differentially expressed genes were presented in the table. I and C represent samples of yeast grown on medium with or without multiple inhibitors in YPD under aerobic condition.

1. **Overexpression of *KmYME* did not improve inhibitor tolerance of *K. marxianus*.**

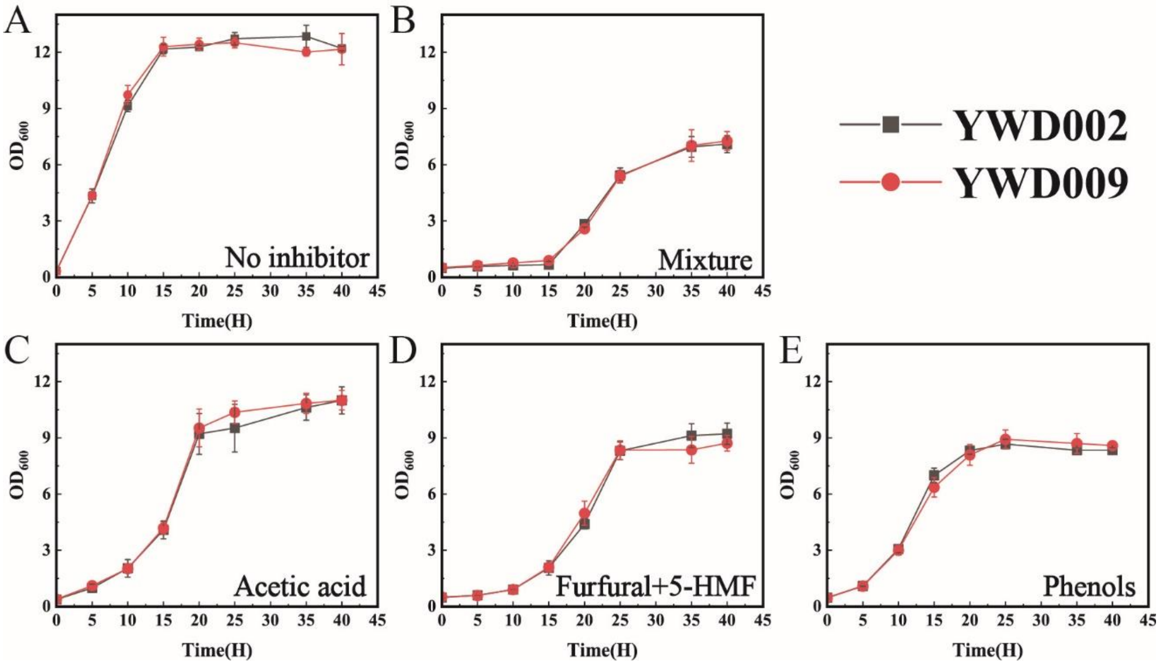

Fig. S1 The growth of YWD002 (*KmYME* overexpressed strain), YWD009 (YHJ010 complemented with *TRP1*, as wild type control) in YPD containing **(A)** no inhibitor, **(B)** inhibitor mixture, **(C)** acetic acid, **(D)** furfural + 5-HMF, or **(E)** phenols. All values are the means of three biological replicates ± standard deviation at each of the time points.

2. **Disruption of *KmYME* decreased yeast tolerance to multiple inhibitors in SD medium.**

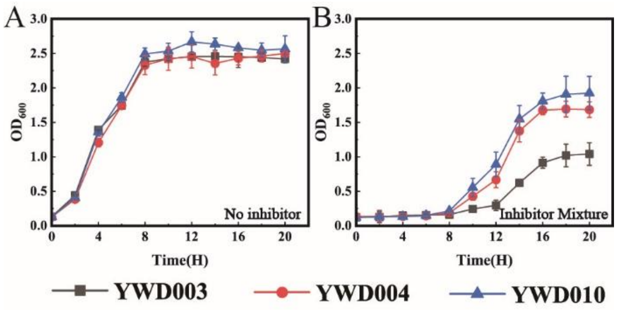

Fig. S2 The growth of YWD003, YWD004, YWD010 cultured in SD medium supplied with tryptophan, leucine and uracil. **(A)** Without inhibitor. **(B)** With inhibitor mixture (1.0 g/l acetic acid, 0.25 g/l furfural, 0.25 g/l 5-HMF, 0.1 g/l phenols). All values are the means of three biological replicates ± standard deviation at each of the time points.

3. **Disruption of *KmYME* led yeast to be more sensitive to acetate and a low pH.**

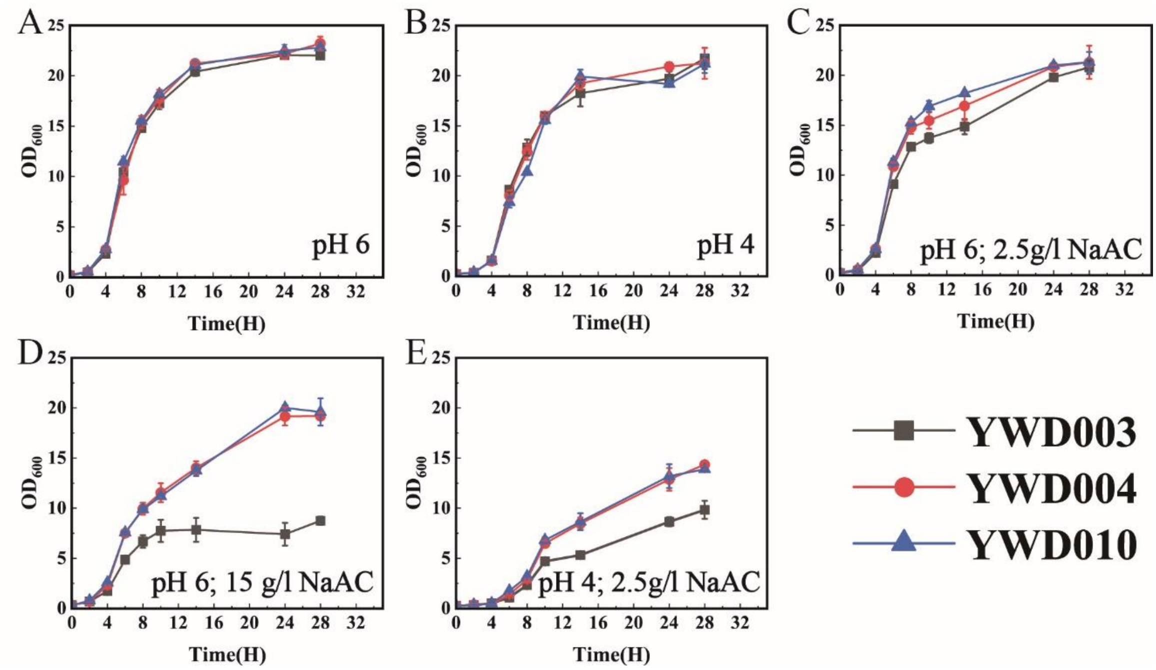

Fig. S3 The growth of YWD003, YWD004 and YWD010 in YPD medium containing **(A)** no acetic acid, pH 6, **(B)** no acetic acid, pH 4, **(C)** 2.5 g/l acetic acid, pH 6, **(D)** 15 g/l acetic acid, pH 6. **(E)** 2.5 g/l acetic acid, pH 4. All values are the means of three biological replicates ± standard deviation at each of the time points.

4. Identify the mitochondrial signal peptide of KmYME.

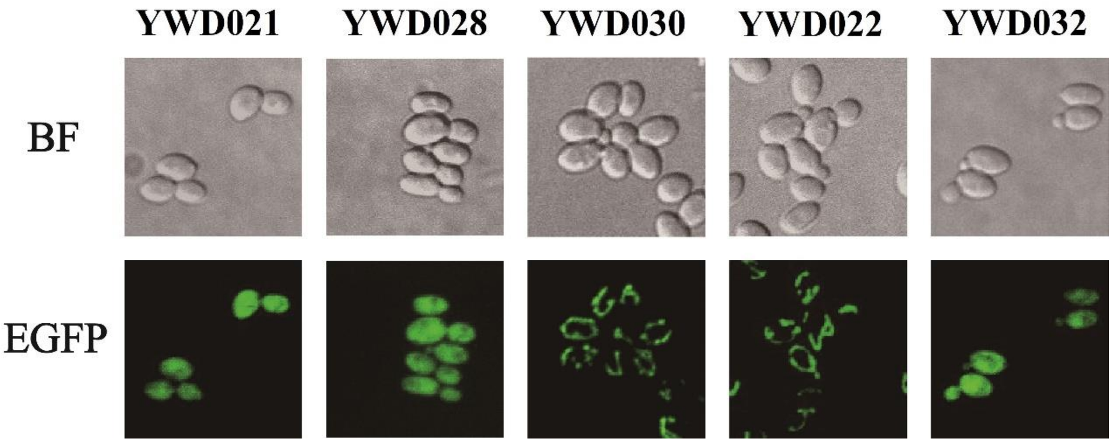

Fig. S4 Identify the mitochondrial signal peptide with truncated KmYME - EGFP. YWD021 (EGFP only), YWD028 (1-20 aa), YWD030 (1-40 aa), YWD022 (1-360 aa, full length), YWD032 (41-360 aa).

5. Mitochondria morphology analysis.

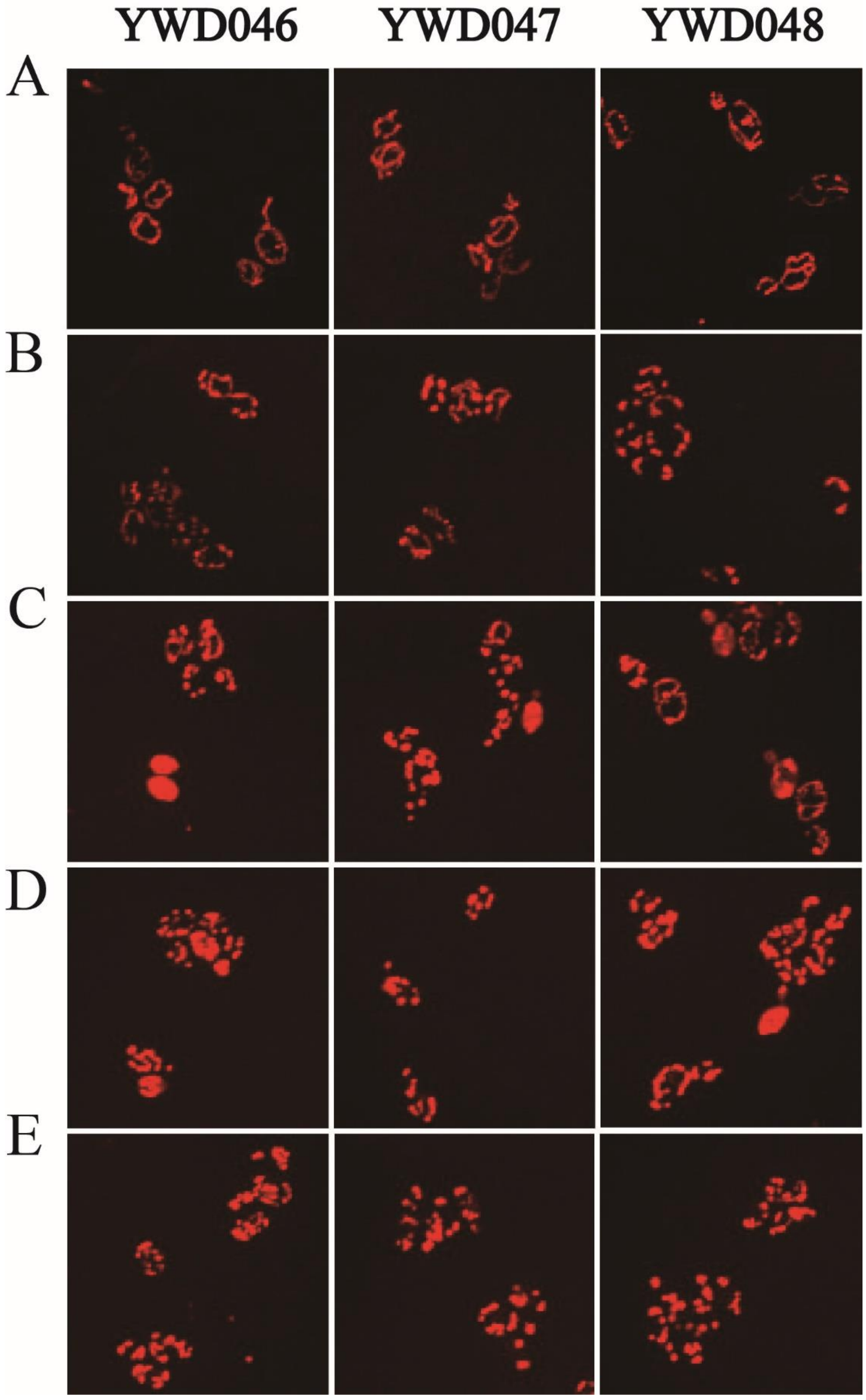

Fig. S5 Mitochondria morphology analysis. YWD046, YWD047 and YWD048 were cultivated in YPD medium containing **(A)** no inhibitors, **(B)** inhibitor mixture, **(C)** acetic acid, **(D)** furfural and 5-HMF, **(E)** phenols.

6. Alignment of amino acid sequences of KmYME and homologous proteins.

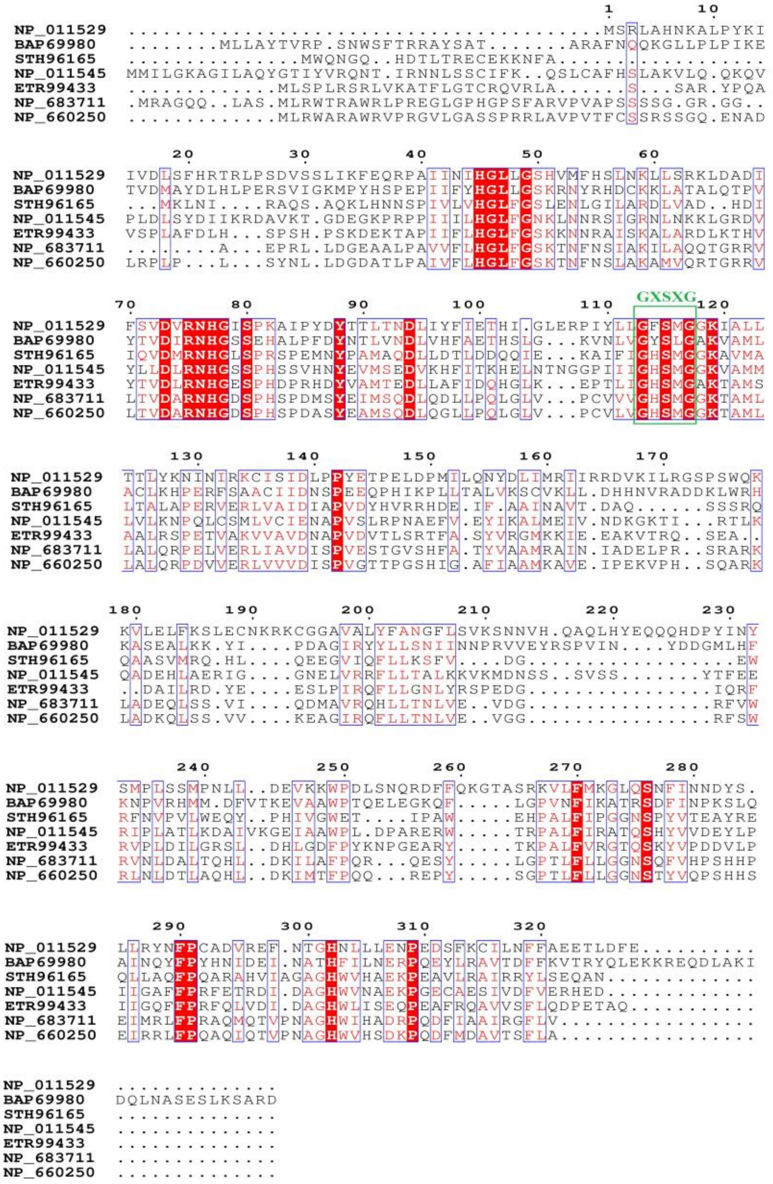

Fig. S6 Alignment of amino acid sequences of KmYME and homologous proteins with common conservative structural domains (GXSXG) (shown in green box) in other species was presented by using a ClustalW web (https://www.genome.jp/tools-bin/clustalw). The order of the species from top to bottom is *Saccharomyces cerevisiae* S288C, *Kluyveromyces marxianus*, *Escherichia coli*, *Saccharomyces cerevisiae* S288C, *Trichoderma reesei* RUT C-30, *Homo sapiens*, and *Mus musculus*, respectively.

7. **SDS-PAGE analysis of purified proteins of KmYME and mutants.**

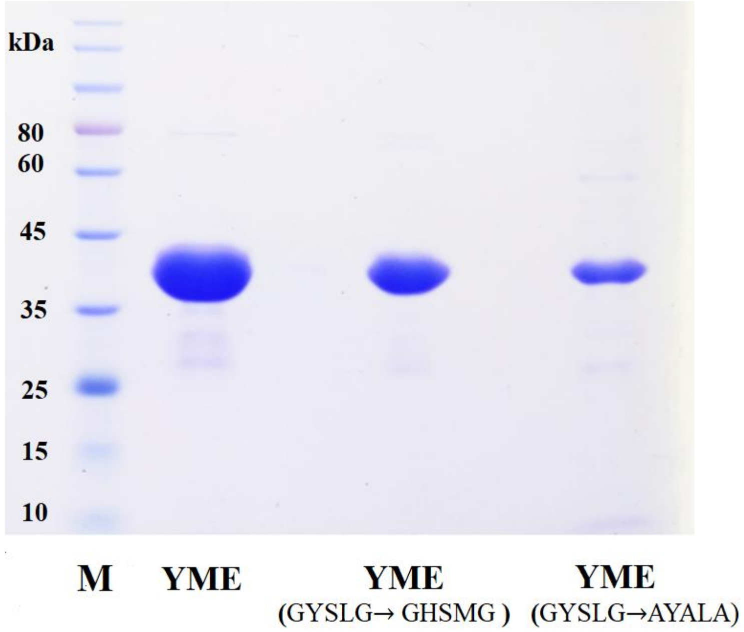

Fig. S7 SDS-PAGE analysis of purified proteins of KmYME and mutants. Molecular weight: about 40 kDa.

8. **Phylogenetic tree of KmYME and possible homologous proteins in other species.**

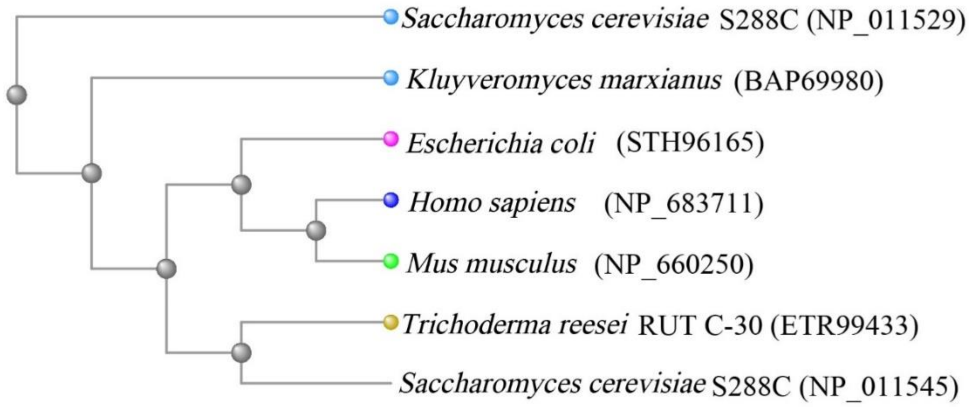

Fig. S8 The phylogenetic tree was established by using a web tool cobalt (https://www.ncbi.nlm.nih.gov/tools/cobalt/).

9. The intracellular CoASH and acetyl-CoA with or without inhibitors.

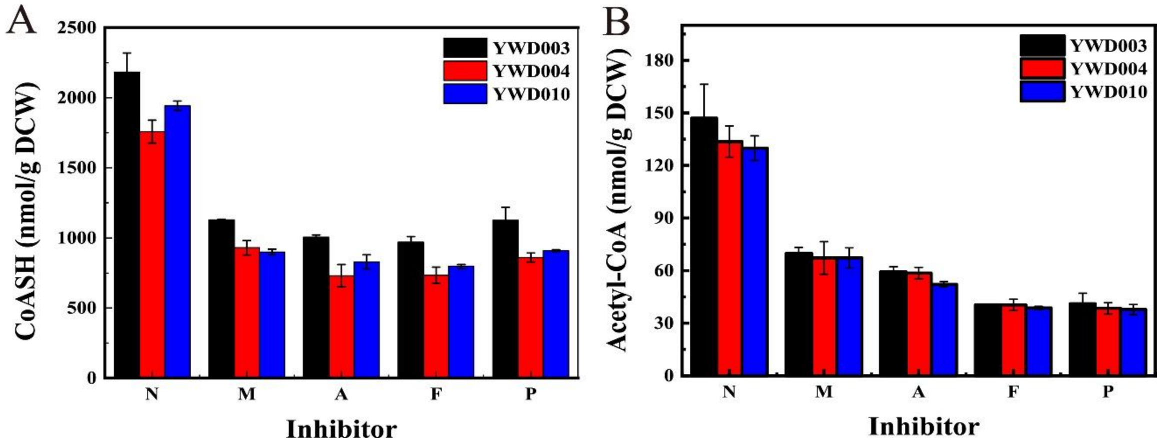

Fig. S9 The intracellular CoASH **(A)** and acetyl-CoA **(B)** with or without inhibitors.
